# Biomimicking trilayer scaffolds with controlled estradiol release for uterine tissue regeneration

**DOI:** 10.1002/EXP.20230141

**Published:** 2024-04-17

**Authors:** Shangsi Chen, Junzhi Li, Liwu Zheng, Jie Huang, Min Wang

**Affiliations:** ^1^ Department of Mechanical Engineering The University of Hong Kong Pokfulam Road Hong Kong China; ^2^ Faculty of Dentistry The University of Hong Kong Sai Ying Pun Hong Kong China; ^3^ Department of Mechanical Engineering University College London London UK

**Keywords:** 3D bioprinting, 4D printing, controlled release, electrospinning, shape morphing

## Abstract

Scaffold‐based tissue engineering provides an efficient approach for repairing uterine tissue defects and restoring fertility. In the current study, a novel trilayer tissue engineering scaffold with high similarity to the uterine tissue in structure was designed and fabricated via 4D printing, electrospinning and 3D bioprinting for uterine regeneration. Highly stretchable poly(l‐lactide‐*co*‐trimethylene carbonate) (PLLA‐*co*‐TMC, “PTMC” in short)/thermoplastic polyurethane (TPU) polymer blend scaffolds were firstly made via 4D printing. To improve the biocompatibility, porous poly(lactic acid‐*co*‐glycolic acid) (PLGA)/gelatin methacryloyl (GelMA) fibers incorporated with polydopamine (PDA) particles were produced on PTMC/TPU scaffolds via electrospinning. Importantly, estradiol (E2) was encapsulated in PDA particles. The bilayer scaffolds thus produced could provide controlled and sustained release of E2. Subsequently, bone marrow derived mesenchymal stem cells (BMSCs) were mixed with gelatin methacryloyl (GelMA)‐based inks and the formulated bioinks were used to fabricate a cell‐laden hydrogel layer on the bilayer scaffolds via 3D bioprinting, forming ultimately biomimicking trilayer scaffolds for uterine tissue regeneration. The trilayer tissue engineering scaffolds thus formed exhibited a shape morphing ability by transforming from the planar shape to tubular structures when immersed in the culture medium at 37°C. The trilayer tissue engineering scaffolds under development would provide new insights for uterine tissue regeneration.

## Graphical abstract

Trilayer scaffolds were fabricated for biomimicking the anatomical structure of the uterine tissue. They could sustainably release estradiol and were able to transform into curved structures to meet the curvature of uterine tissue. They therefore have a high potential for uterine regeneration.

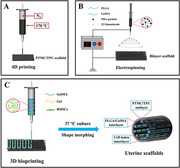

## 1 INTRODUCTION

The uterus in females provides essential biological functions in human reproduction, such as the implantation and growth of embryos. Congenital anomalies and acquired diseases caused by intrauterine adhesion (IUA), infection or hysteromyoma may lead to uterus dysfunction and hence compromise a woman's ability to be pregnant and/or carry a healthy fetus to term.^[^ ^1^ ^]^ Currently, infertility is a severe problem in our society. Infertile male‐and‐female couples at reproductive ages suffer significantly from both emotional and mental problems. About 0.2% of women have been diagnosed with absolute uterine factor infertility (AUFI) and approximately 6% of them need uterine repair treatments.^[^ ^2^ ^]^ Fortunately, the first birth of a healthy child following uterus transplantation was reported in the United States in 2018,^[^ ^3^ ^]^ giving infertile couples a glimpse of hope. Afterwards, successful cases have been continuously reported about women giving birth to healthy children after receiving a transplanted uterus.^[^ ^4^ ^]^ Although uterus transplantation has been an effective treatment for AUFI, the problems of donor shortage, possible disease transmission and use of antirejection drugs limit its wide applications. Therefore, new solutions to regenerate the structure and restore the functions of the injured uterus need to be found or developed.

Tissue engineering has been shown to be a promising approach to repair damaged tissues/organs in the body by using biological substitutes by combining three‐dimensional (3D) scaffolds, cells/stem cells and biomolecules and therefore overcome the hurdles in tissue or organ transplantation.^[^ ^5^ ^]^ Tissue engineering has already made great success in treating problems for various tissues and organs, including bone,^[^ ^6^ ^]^ blood vessel,^[^ ^7^ ^]^ skin,^[^ ^8^ ^]^ and bladder.^[^ ^9^ ^]^ Several types of natural or synthetic biodegradable polymer scaffolds encapsulated with appropriate stem cells or biomolecules have been made and studied for repairing damaged uterine tissue in vitro or in vivo, owing to their good biocompatibility and biodegradability.^[^ ^10^ ^]^ For example, collagen‐based scaffolds are an attractive option for uterine regeneration.^[^ ^11^ ^]^ Ding et al. showed that a 3D collagen membrane loaded with bone marrow‐derived mesenchymal stem cells (BMSCs) promoted the healing of severe uterine injuries in rats.^[^ ^12^ ^]^ They found that collagen/BMSC constructs facilitated the proliferative abilities of the uterine endometrial and muscular cells and restored the ability of the endometrium to receive an embryo and support its development to a viable stage. However, the progress of using tissue engineering scaffolds for uterine regeneration is still very limited. Most studies in this area have remained in the preclinical stage.^[^ ^5^, ^13^ ^]^ There is a lack of clinical studies to examine the therapeutic efficacy of these tissue engineering scaffolds for uterine tissue regeneration.

On the other hand, another deficiency is that the current efforts for uterine treatments appear to have centered on endometrium regeneration.^[^ ^14^ ^]^ Indeed, endometrium in the uterus is essential for embryo implantation and pregnancy maintenance because of its dynamic remodeling process and significant regenerative capacity. The damage to endometrium does cause infertility. But the uterine tissue has a hierarchical structure and consists of three layers: outlayer of the perimetrium, interlayer of the myometrium and the innerlayer of endometrium. Endometrium consists of a basal layer and a functional layer. Myometrium, which contains uterine smooth muscle cells, plays an important role in inducing uterine contraction and supporting stromal and vascular tissues. An injury of myometrium also significantly affects the uterine structure and functions and further results in infertility.^[^ ^15^ ^]^ In this context, the reported studies focusing on endometrium repair may have indicated, to some extent, the inability to restore or regenerate the structure and functions of a whole, multilayered uterine tissue. Additionally, although a recent study reported the construction of a tissue‐engineered uterine scaffold seeded with autologous stem cells to support live births in rabbit,^[^ ^1^ ^]^ the scaffold had limited elasticity, which would hinder its further applications because the human uterus has excellent stretchability property. Therefore, the development of tissue engineering scaffolds possessing a multilayered structure and high elasticity to mimic the inherent structure and properties of the natural uterus would be significantly beneficial for uterine tissue regeneration.

**FIGURE 1:**
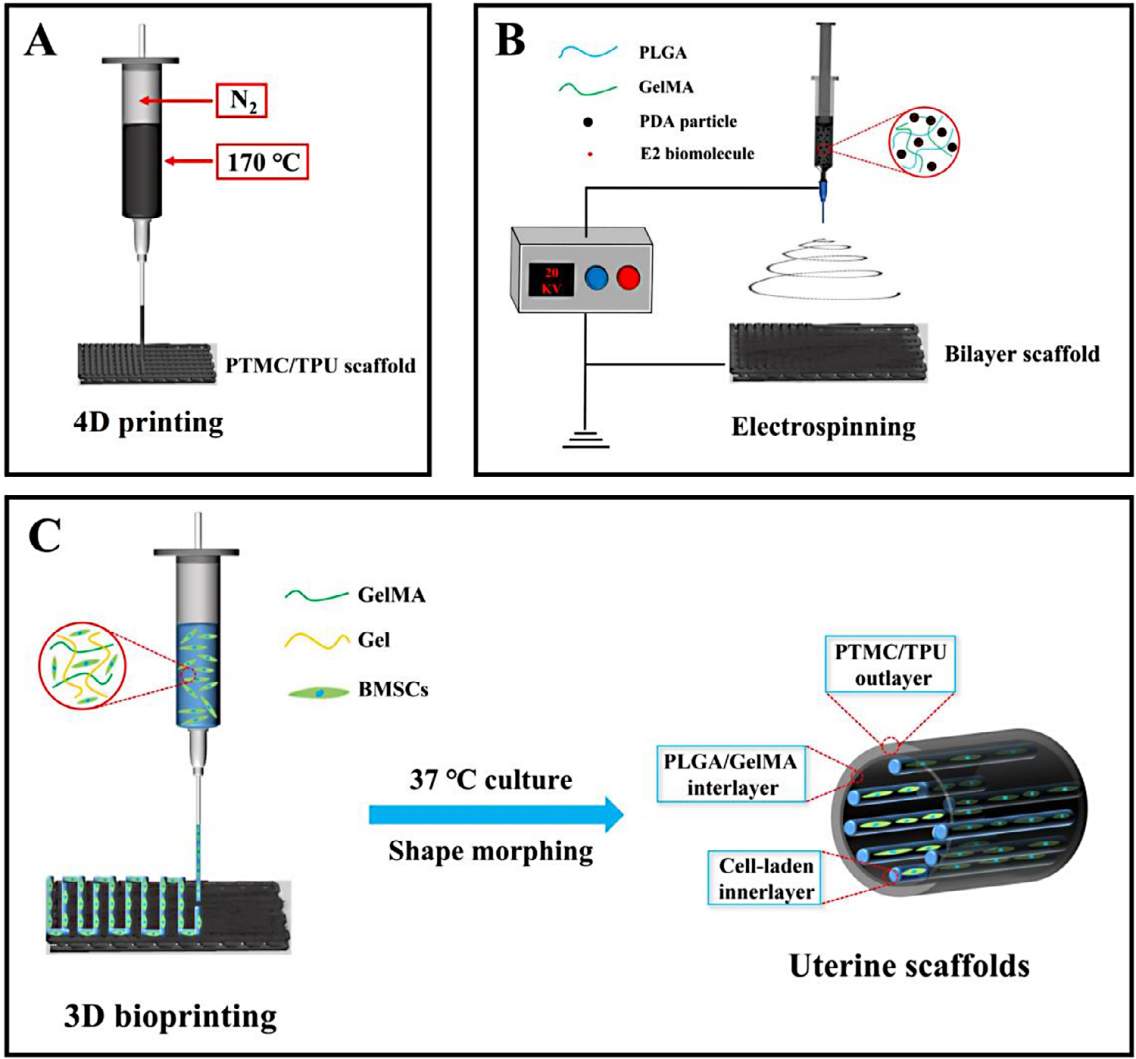
Schematic illustration for the fabrication of trilayer tissue engineering scaffolds for uterine tissue regeneration. (A) 4D printing of the PLLA‐*co*‐TMC (PTMC)/thermoplastic polyurethane (TPU) scaffold layer. (B) Electrospinning to fabricate the E2‐containing poly(lactic acid‐*co*‐glycolic acid) (PLGA)/gelatin methacryloyl (GelMA) fibrous layer on the PTMC/TPU scaffold layer. (C) 3D bioprinting to construct the BMSC‐laden hydrogel layer on the PLGA/GelMA fibrous layer to complete the fabrication of designed biomimicking trilayer tissue engineering scaffold.

3D printing has been extensively investigated in the tissue engineering field because of its great ability to manufacture objects of complex shapes and structures, as well as products of customized designs.^[^ ^16^ ^]^ To overcome the drawbacks (mainly, in relation to time, static shapes, properties or functions of printed objects) of 3D printed structures, 4D printing emerged recently, for which time as the fourth dimension was integrated with 3D printing.^[^ ^17^ ^]^ On the other hand, 3D bioprinting is now increasingly used for fabricating cell‐laden tissue engineering scaffolds.^[^ ^18^ ^]^ In the current study, a tissue engineering scaffold mimicking the structure and properties of native uterine tissue with a multilayered structure and high elasticity was designed and scaffolds of this design were fabricated via 4D printing, electrospinning and 3D bioprinting (Figure 1). Firstly, to mimic the highly stretchable myometrium layer of the uterine tissue, poly(l‐lactide‐*co*‐trimethylene carbonate) (PLLA‐*co*‐TMC, “PTMC” in short) and thermoplastic polyurethane (TPU) were homogenously mixed to fabricate the PTMC/TPU scaffold layer via fused deposition modeling (FDM). PTMC/TPU scaffolds thus produced exhibited high stretchability. Next, a poly(lactic acid‐*co*‐glycolic acid) (PLGA) and gelatin methacryloyl (GelMA) mixed solution was employed to fabricate a PLGA/GelMA fibrous layer on the PTMC/TPU scaffold through electrospinning for mimicking functions of the basal layer of endometrium. Estradiol (E2), an essential steroid hormone, was encapsulated in the PLGA/GelMA fibrous layer. E2 is a clinically used biomolecule for promoting uterine regeneration by binding to its receptors and then activating the expression of various angiogenic growth factors. E2 could be controllably and sustainably released to regulate cell behavior. Furthermore, BMSC‐laden GelMA/Gel hydrogel was 3D bioprinted on the PLGA/GelMA fibrous layer to mimic the functions of the functional layer of endometrium, thereby forming the complete trilayer tissue engineering scaffolds. BMSCs showed a very high survival rate and were homogenously distributed in the 3D bioprinted hydrogel layer. The trilayer scaffolds possessed a layered structure similar to that of the human uterus and were highly elastic. Moreover, the trilayer scaffolds could evolve from a planar shape to tubular structures when cultured at 37°C. Therefore, these trilayer tissue engineering scaffolds with a hierarchical structure, high stretchability, and controlled and sustained E2 release would have a high potential for uterine tissue regeneration.

## 2 MATERIALS AND METHODS

### 2.1 Materials

PTMC (molar ratio of LA:TMC = 8:2 and 0.9 dl g^−1^) and PLGA (molar ratio of LA:GA = 50:50 and average molecular weight of 90 kDa) were purchased from Jinan Daigang Ltd., China. TPU 95A was supplied by BASF SE, Germany. Estradiol (E2) was bought from Aladdin Co. Ltd., China. Gelatin (porcine skin, type A), bovine serum albumin (BSA), methacrylate anhydride (MA, 94%), ammonium hydroxide solution (NH_4_OH) (28%), absolute ethanol, 2‐hydroxy‐2‐methylpropiophenone (97%), 1,1,1,3,3‐hexafluoro‐2‐propanol (HFIP) and dopamine hydrochloride (98%) were bought from Sigma‐Aldrich (St. Louis, MO, USA). Dialysis tubing cellulose membrane (MWCO 10 kDa) was supplied by Thermo Fisher Scientific, USA. All reagents were used as received without further purification.

### 2.2 Fabrication and characterization of PTMC/TPU scaffolds

Different amounts of PTMC and TPU at specific PTMC:TPU ratios were homogeneously mixed in round‐bottom flasks by mechanical stirring after being heated in an oil bath to 170°C under nitrogen protection. Afterward, PTMC/TPU mixtures were cut into pieces and then transferred to a stainless steel cartridge in a 3D bioprinter (regenHU, Switzerland). 4D printing was conducted using these printing parameters: the inner diameter of the printing nozzle was 410 µm, the printing temperature was 170°C, and the printing speed was 8 mm s^−1^. Subsequently, 4D printed PTMC/TPU scaffolds (designated as S scaffolds) were rolled up to tubular structures using a stainless steel rod (rod diameter: 8 mm) in an oven at 80°C for 30 min. The tubular scaffolds were then flattened at room temperature for further use.

A scanning electron microscope (SEM, Hitachi S‐4800, Japan) was used to observe the surface morphology and microstructure of 4D printed PTMC/TPU scaffolds. Differential scanning calorimetry (DSC, Pyris 6, PerkinElmer, USA) was conducted to analyze the glass transition temperature of PTMC/TPU scaffolds, and thermogravimetric analysis (TGA, DZ‐TGA101, Nanjing Dazhan Testing Instrument Co., Ltd, China) was performed to determine the real PTMC:TPU ratios for PTMC/TPU scaffolds. The mechanical properties of PTMC/TPU scaffolds were studied via tensile tests using a universal testing machine (Model 5848, Instron Ltd., USA) at room temperature and at 37°C, respectively. The surface wettability and BSA adsorption of PTMC/TPU scaffolds were studied. Moreover, the shape morphing behavior of PTMC/TPU scaffolds was investigated by immersing scaffolds in a water bath at 37°C, with shape‐morphing processes being recorded by a digital camera.

### 2.3 Synthesis of GelMA, PDA and PDA@E2 particles

GelMA synthesis was similar to previous studies.^[^ ^19^ ^]^ Briefly, gelatin (10 g) was dissolved in phosphate buffered saline (PBS, 100 mL, 0.01 m, pH 7.4) at 50°C. MA (0.8 mL) was added dropwise into the gelatin solution. After a reaction for 1 h, PBS (500 mL) was added to stop the reaction and the obtained solution was transferred into dialysis tubes to dialyze against deionized (DI) water for 7 days at 40°C. The DI water was refreshed daily. GelMA was obtained after lyophilization. To quantify the degree of substitution of GelMA, ^1^H‐NMR spectroscopy (Bruker Avance III 400, USA) analysis was conducted.

The synthesis of PDA and PDA@E2 particles followed previously reported procedures^[^ ^20^ ^]^ but with some small modifications. Because E2 is poorly water‐soluble, a water/ethanol mixture was used to synthesize PDA@E2 particles. Different amounts of E2 (0 or 0.75 g) were dissolved in absolute ethanol under constant magnetic stirring for at least 2 h. Ammonia (10 mL) and DI water (290 mL) were added into ethanol solution under stirring for 30 min. Subsequently, 50 mL of 5 w v^−1^% dopamine hydrochloride solution was poured into the ethanol/water mixture under constant stirring for 24 h at room temperature in open air. PDA and PDA@E2 particles were obtained after centrifugation at 12,000 rpm min^−1^ for 10 min. The morphology and microstructure of PDA and PDA@E2 particles were characterized using SEM and a transmission electron microscope (TEM, Tecnai G2 20, USA). The diameter distribution of particles was analyzed using Image J software.

### 2.4 Fabrication of PLGA/GelMA fibers on PTMC/TPU scaffolds

PLGA (15 w v^−1^%), GelMA (5 w v^−1^%) and 2‐hydroxy‐2‐methylpropiophenone (0.5 w v^−1^%) were dissolved in HFIP solvent to prepare a PLGA/GelMA solution for electrospinning. The solution was then transferred to a 10 mL syringe equipped with a steel needle with an inner diameter of 0.8 mm. The syringe was fixed to a pump (LongerPump, LSP01‐3A, UK). The steel needle was connected to a high‐voltage power supply (Kou Hing Hong Scientific Supplies Ltd., HK). To determine the optimal parameters for electrospinning of PLGA/GelMA fibers (designated as F scaffold in bilayer scaffolds), different applied voltages (10, 15, 20 kV) and feeding rates (0.5, 1.0, 2.0 mL h^−1^) were investigated. The 3D‐printed PTMC/TPU scaffolds were attached to aluminum foil. The aluminum foil was connected to the high‐voltage power supply and placed 10 cm away from the needle tip to collect electrospun PLGA/GelMA fibers on the PTMC/TPU scaffolds. The bilayer scaffolds thus formed were immersed in absolute ethanol and then exposed to UV light (365 nm) for 10 min. Subsequently, they were incubated in an oven at 50°C overnight to totally remove ethanol and make the PTMC/TPU scaffold layer and electrospun fiber layer attached to each other tightly. In the current study, these bilayer scaffolds were designated as S+F scaffolds. The fabrication of S+F‐PDA scaffolds was similar to the above process but with different concentrations (0.5%, 1.0%, 2.5% and 5.0%, with the concentration referring to the PDA mass to the total mass of PLGA and GelMA) of PDA particles dispersed in the PLGA/GelMA electrospinning solution.

The surface morphology and diameters of electrospun PLGA/GelMA fibers and PLGA/GelMA‐PDA (designated as F‐PDA) fibers were studied using SEM. The mechanical properties of PLGA/GelMA and PLGA/GelMA‐PDA fibers were investigated via tensile tests. Moreover, the surface and cross‐sectional morphology of S+F‐PDA scaffolds were examined through SEM. The mechanical performance and shape morphing behavior of bilayer scaffolds were also investigated.

### 2.5 Evaluation of photothermal effect and controlled E2 release from bilayer scaffolds

The encapsulation of PDA particles in PLGA/GelMA fibers would produce a photothermal effect for the scaffolds. To study the photothermal effect, bilayer S+F‐PDA scaffolds were exposed to a near‐infrared (NIR) laser (808 nm wavelength) at different densities (0.5 and 1.0 W cm^−2^). The temperature changes were revealed by thermal images, which were recorded using an infrared camera (GUIDE EasIR‐9, AutoNavi, China). Additionally, to study the effect of NIR laser irradiation on E2 release, S+F‐PDA@E2 scaffolds were exposed to different power densities (0, 0.5, and 1.0 W cm^−2^) of the NIR laser for 30 min every 1 hour in PBS (pH 7.4) at 37°C. PBS was collected at each predetermined time point, and an equal amount of fresh PBS was added to the system. The amount of E2 released in PBS was determined using a UV–vis spectrophotometer (UV‐2600, Shimadzu, Japan) at the wavelength of 280 nm, and cumulative release curves for E2 were then plotted.

Since the human uterus has a dynamic pH environment, it is very important to investigate E2 release kinetics in solutions of different pH values. S+F‐PDA@E2 scaffold samples were therefore immersed in different buffer solutions (at pH 4.5, pH 7.4, and pH 9.0) at 37°C. At each predetermined time point, a small amount of buffer solution was extracted from the release system, and an equal amount of fresh buffer solution was supplemented. The amount of E2 released was determined using the UV–vis spectrophotometer. The cumulative amounts of E2 released in different buffer solutions were calculated, and E2 release curves were established.

### 2.6 In vitro biological evaluation of bilayer scaffolds

To evaluate the biological properties of bilayer scaffolds, BMSCs extracted from healthy adult rats were used in in vitro experiments. BMSCs were propagated in high‐glucose Dulbecco's modified Eagle's medium (DMEM, Gibco) with 10% (v v^−1^) fetal bovine serum (FBS, Gibco) and 1% (v v^−1^) penicillin‐streptomycin (Gibco). The cell culture work was performed in a CO_2_ incubator supplemented with 5% CO_2_ and 95% humidity at 37°C.

The attachment, survival, proliferation and morphology of BMSCs on PTMC/TPU (designated as “S”), S+F and S+F‐PDA@E2 scaffolds were studied through SEM observation, live/dead assays (Invitrogen, Thermo Fisher Scientific), MTT assays (Invitrogen, Thermo Fisher Scientific), and confocal laser scanning microscopy (LCSM, Leica, Germany) after phalloidin/DAPI staining (Invitrogen, Thermo Fisher Scientific), respectively. BMSCs at a density of 5 × 10^4^ cells per well were seeded on S, S+F and S+F‐PDA@E2 scaffold samples in a 24‐well cell culture plate and cultured in DMEM at 37°C. After being immobilized by 4% paraformaldehyde for 30 min, samples were dehydrated by a serial gradient ethanol solution (50%, 60%, 70%, 80%, 90%, and 100%) for 10 min and then dried in a vacuum oven overnight. The dried samples were then sputtered with a thin layer of gold, and cell morphology was observed under SEM.

For cell survival analysis, BMSCs at the density of 5 × 10^4^ cells per well were seeded on S, S+F and S+F@‐PDAE2 scaffolds in a 24‐well cell culture plate and cultured in DMEM at 37°C. After culture for 24 and 48 h, respectively, a live/dead assay was used to stain BMSCs. Living cells were stained green, and dead cells were stained red when observed under a fluorescence microscope (Leica DMi8, Germany). Moreover, the cell survival rate was calculated using Image J software. On the other hand, BMSCs at a density of 1 × 10^4^ cells per well were seeded on scaffold samples to study the BMSC proliferation rate and cell morphology. After culture for 1, 3 and 7 days, respectively, BMSC viability on S, S+F and S+F‐PDA@E2 scaffolds was determined using the MTT assay. Furthermore, BMSCs were visualized under CLSM after phalloidin/DAPI staining.

### 2.7 Rheological properties of BMSC‐laden GelMA/Gel bioinks

GelMA (7 w v^−1^%), gelatin (3 w v^−1^%) and BMSCs at a density of 1 × 10^6^ per mL were mixed to prepare BMSC‐laden GelMA/Gel bioinks for 3D bioprinting. The rheological properties of GelMA/Gel inks and BMSC‐laden GelMA/Gel bioinks were investigated at 20°C using a rheometer (MCR 302, 176 Anton Paar, Austria) equipped with a parallel plate unit with a 20 mm diameter. In accordance with the preliminary strain sweep test results, GelMA/Gel inks and BMSC‐laden GelMA/Gel bioinks with or without UV crosslinking were loaded onto a parallel plate and subjected to a maximum strain (γ) of 1.0% under continuous oscillation. In the frequency sweep mode, the storage moduli (*G*′) and loss moduli (*G*ʺ) of GelMA/Gel inks and BMSC‐laden GelMA/Gel bioinks were measured in the range of 0.1–100 rad s^−1^. Furthermore, the shear thinning behavior of GelMA/Gel inks and BMSC‐laden GelMA/Gel bioinks was studied. The viscosity of each sample was measured over the temperature range of 15–50°C at a 1 s^−1^ shear rate. Thixotropic tests were conducted in two repeated steps by varying the shear rate. First, a low shear rate (1 s^−1^) was applied for 120 s. Then, a high shear rate (500 s^−1^) was applied for 60 s.

### 2.8 Fabrication of the complete biomimicking trilayer tissue engineering scaffolds

BMSC‐laden GelMA/Gel bioinks were used to fabricate the cell‐laden hydrogel layer on S+F‐PDA@E2 bilayer scaffolds via 3D bioprinting for the construction of designed biomimicking trilayer tissue engineering scaffolds. In the current study, GelMA/Gel inks were homogeneously mixed with BMSCs at room temperature. The resulting cell‐laden bioinks were subsequently transferred to a cartridge in a 3D bioprinter. The printing parameters were set as follows: the inner diameter of the printing nozzle was 210 µm, the printing temperature was 20°C, and the printing speed was 6 mm s^−1^. After adjusting the height between the S+F‐PDA@E2 scaffold and printing nozzle, BMSC‐laden GelMA/Gel hydrogels were 3D printed on S+F‐PDA@E2 scaffolds. Subsequently, UV light (wavelength: 365 nm; power: 365 mW) was used to crosslink the hydrogel layer for 2 min. The trilayer tissue engineering scaffolds thus produced were transferred to a 6‐well cell culture plate and cultured with DMEM at 37°C. The printability of BMSC‐laden GelMA/Gel bioinks was evaluated using the following formula:

(1) $$\mathrm{Printability}=L^{2}/16A$$

where *L* is the perimeter and *A* is the area of the pore. If the printability value is close to 1, it signifies the square shape of the pores, suggesting good printability of the ink or bioink.^[^ ^21^ ^]^

### 2.9 Cell survival rate and shape morphing of trilayer tissue engineering scaffolds

Live/dead assay was used to examine the BMSC survival rate in the GelMA/Gel hydrogel layer. After printing and then culturing for 1, 3 and 7 days, respectively, BMSCs in the printed hydrogel layer were stained by live/dead assay according to the manufacturer's protocol. Additionally, the BMSC distribution in the final trilayer tissue engineering scaffolds was visualized via CLSM. The shape morphing behavior of trilayer scaffolds was recorded using a digital camera when scaffolds were immersed in the culture medium at 37°C.

### 2.10 Statistical analysis

The results presented in this article were obtained from at least three separate samples and were expressed as the mean ± SD. One‐way ANOVA was performed for statistical analysis. Statistically significant difference existed when: *p < 0.05, **p < 0.01, and ***p < 0.001.

## 3 RESULTS AND DISCUSSION

### 3.1 Fabrication, structure and properties of PTMC/TPU scaffolds

**FIGURE 2:**
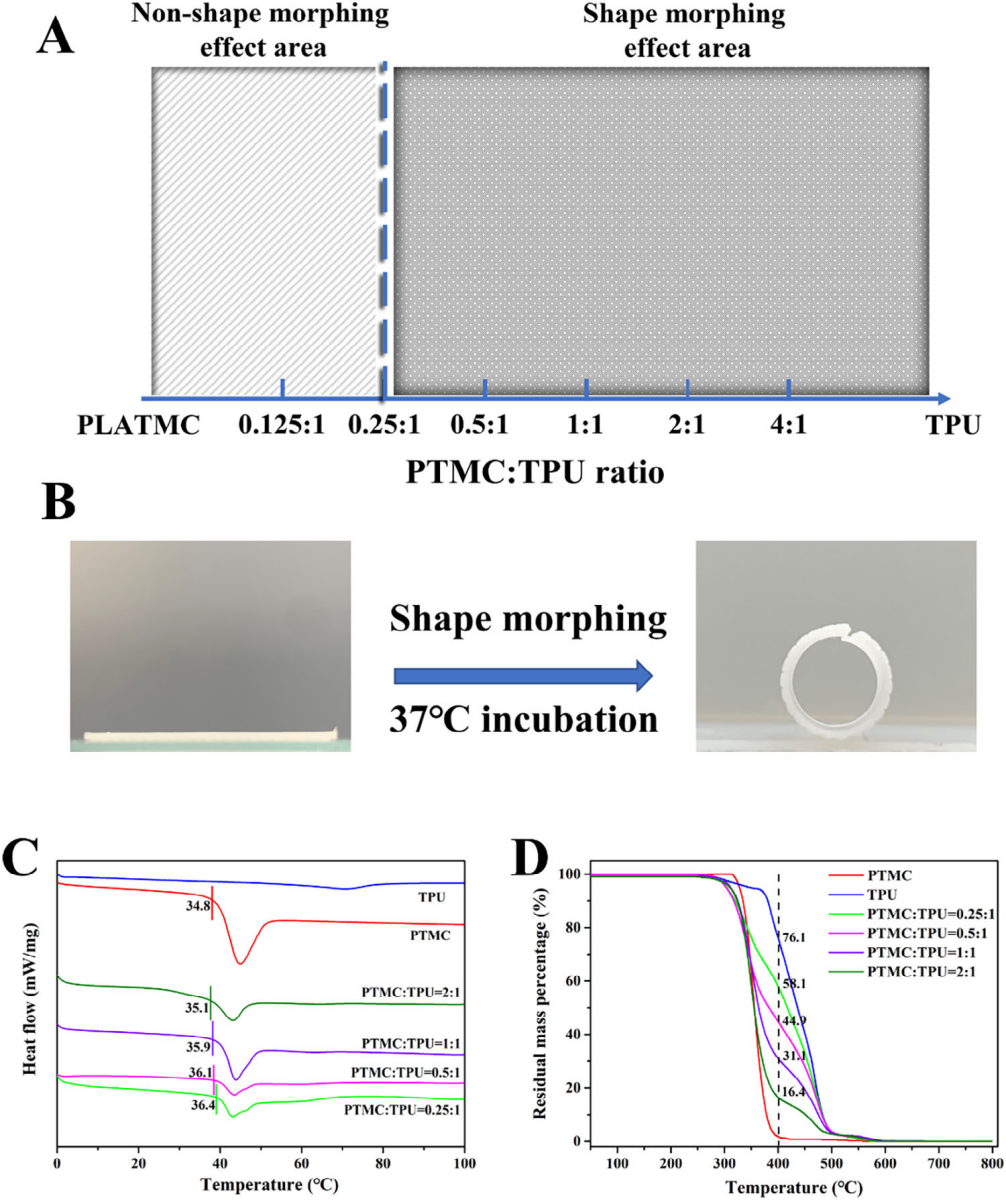
(A) A diagram illustrating the effect of the PTMC:TPU ratio on the shape morphing ability of PTMC/TPU scaffolds. (B) Shape morphing behavior of PTMC/TPU scaffolds (PTMC:TPU of 0.25:1) when immersed in the culture medium at 37°C. (C) DSC curves and (D) TGA results of 3D printed PTMC/TPU scaffolds.

The high stretchability of the human uterus is mainly due to the intrinsic elasticity of the myometrium. Elastomeric TPU has been extensively used to restore damaged vascular and skeletal muscles.^[^ ^22^ ^]^ Moreover, TPU elastomers possess controllable mechanical properties that can be used to match the targeted body tissues.^[^ ^23^ ^]^ In the current study, to mimic the functions and properties of myometrium, TPU, as a highly elastic material, is a good biomaterial for scaffold fabrication. On the other hand, given the curved shape of the uterus, it is important to fabricate scaffolds that have the shape morphing ability to form a curved or tubular shape to match the curvature of the repair site in the uterus. Previous studies indicated that biodegradable PTMC polymers exhibited programmed shape morphing from a planar shape to a tubular structure after being incubated at human body temperature (37°C).^[^ ^24^ ^]^ Therefore, in the current study, PTMC was chosen to mix homogeneously with TPU for fabricating PTMC/TPU scaffolds via FDM. To determine the optimal properties of PTMC/TPU scaffolds (i.e., comparable mechanical strength with the uterus and good shape‐morphing ability), mixtures of different PTMC:TPU ratios were made under mechanical stirring at 170°C. Subsequently, PTMC/TPU mixtures were 3D printed to form polymer blend scaffolds. As shown in Figure 2A and Figure S1, when the PTMC:TPU ratio was below 0.25:1, PTMC/TPU scaffolds were unable to transform from planar shapes to tubular structures after immersion in water at 37°C. On the contrary, at higher PTMC:TPU ratios, PTMC/TPU scaffolds exhibited shape morphing ability (Figure 2B, Video S1). The shape memory effect of PTMC/TPU scaffolds could be attributed to the suitable glass transition (*T*_g_) temperature of PTMC. DSC results shown in Figure 2C indicated that the *T*_g_ temperatures of PTMC and PTMC/TPU scaffolds were at about 36°C. The shape morphing behavior of these scaffolds could be attributed to the amorphous nature of the PTMC polymer. After 3D printing, scaffolds were shaped into tubular structures using a stainless‐steel rod in an oven at 80°C for 30 min. The gradient increase in temperature from the surface to the interior during the heating process affected the degree of molecular orientation, thereby resulting in anisotropic birefringence and inhomogeneous transparency. Therefore, PTMC/TPU polymer blend scaffolds could retain their temporary planar shape at room temperature and completely recover their permanent tubular structure at 37°C when the glass transition of PTMC/TPU scaffolds started to take place. Furthermore, TGA results (Figure 2D and Table S1) showed that the real PTMC percentage in PTMC/TPU scaffolds was almost the same as the nominal percentage, suggesting that PTMC and TPU were homogenously mixed under mechanical stirring at 170°C. It was revealed by DTG curves (Figure S2) that PTMC/TPU scaffolds at all PTMC:TPU ratios had similar thermal degradation behavior.

**FIGURE 3:**
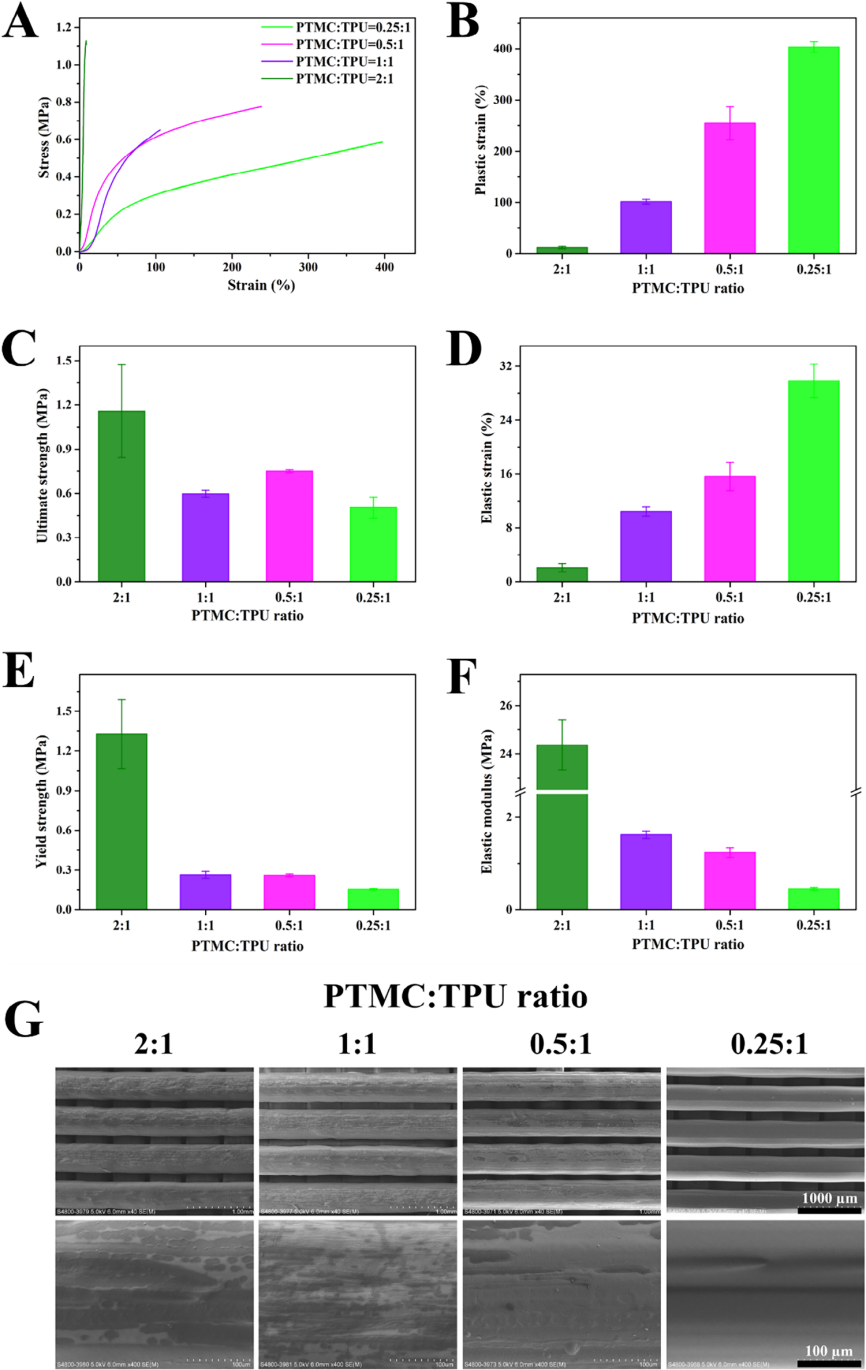
Surface morphology and mechanical properties of 3D printed PTMC/TPU scaffolds. (A) Tensile stress–strain curves, (B) plastic strain, (C) ultimate tensile strength, (D) elastic strain, (E) yield strength and (F) elastic modulus of 3D printed PTMC/TPU scaffolds. (G) SEM images showing the surface morphology of PTMC/TPU scaffolds.

Since the PTMC/TPU scaffolds were made to mimic the functions of myometrium, suitable mechanical properties from these scaffolds are essential. Previous *ex vivo* studies indicated that the ultimate tensile strength of porcine uterine tissues was 320 ± 176 kPa with a corresponding strain of 30 ± 9.0% and that the human uterus exhibited better mechanical performance with an average ultimate strength of 656.3  ±  483.9 kPa at a strain of 32 ± 11.2%.^[^ ^25^ ^]^ Another study also claimed that the mechanical strength of native uterine tissues was 0.258 ± 0.071 MPa.^[^ ^26^ ^]^ Moreover, in vivo tensile tests suggested that the strain of the human uterus could be up to 110–130%.^[^ ^27^ ^]^ In this context, for adequately biomimicking the mechanical performance of the uterus, PTMC/TPU scaffolds should have an ultimate strength of over 200 KPa and a strain over 100% at the human body temperature. In the current study, the mechanical strength of PTMC/TPU scaffolds was measured via tensile tests at room temperature and at human body temperature, respectively. Figure 3A–F shows the mechanical behavior and properties of PTMC/TPU scaffolds at room temperature. When the PTMC:TPU ratio was 2:1, PTMC/TPU scaffolds had a high ultimate strength of 1.16 ± 0.31 MPa but a very low strain of 11.52 ± 2.27%, which was not suitable for mimicking the functions of the human uterus. When the PTMC:TPU ratio was at 1:1, 0.5:1 and 0.25:1, PTMC/TPU scaffolds possessed the desirable mechanical strength of ≈0.6 MPa and an appropriate strain of over 100%. Particularly for scaffolds at the PTMC:TPU ratio of 0.25:1, the strain was about 400%. Because the glass transition temperatures of PTMC/TPU scaffolds were below 37°C, these scaffolds would become more elastic at the human body temperature. As shown in Figure S3 for tests conducted at 37°C, the strain of PTMC/TPU scaffolds dramatically increased, while the mechanical strength of PTMC/TPU scaffolds decreased. The highest mechanical strength of PTMC/TPU scaffolds (at the PTMC:TPU ratio of 0.25:1) was 0.25 ± 0.02 MPa at 37°C. Consequently, owing to their comparable mechanical strength to native uterine tissues, PTMC/TPU scaffolds at a PTMC:TPU ratio of 0.25:1 were used in subsequent experiments.

The surface morphology of 3D printed PTMC/TPU scaffolds is displayed in Figure 3G, showing that scaffolds at different PTMC:TPU ratios all had relatively smooth surfaces, especially for scaffolds at the PTMC:TPU ratio of 0.25:1. The smooth surface makes scaffolds less hydrophilic, which is unfavorable for cells, including their adhesion, proliferation and differentiation.^[^ ^28^ ^]^ Additionally, due to the high content of TPU, PTMC/TPU (0.25:1) scaffolds had a hydrophobic surface and the water contact angle was 112.3 ± 6.3° (Figure S4A). The BSA adsorption experiment also indicated that PTMC/TPU (0.25:1) scaffolds had the lowest BSA adsorption amount (Figure S4B), suggesting poor surface properties of PTMC/TPU (0.25:1) scaffolds.

### 3.2 Fabrication, structure and properties of bilayer scaffolds

The hydrophobic surface of a scaffold is not beneficial for scaffold‐cell interactions and can cause poor tissue regeneration outcomes.^[^ ^29^ ^]^ To improve surface properties, PLGA/GelMA fibers were constructed on PTMC/TPU scaffolds via electrospinning. PLGA and GelMA are two popular biomaterials used in tissue engineering.^[^ ^30^ ^]^ Previous studies by a few groups employed PLGA and GelMA scaffolds or hydrogels to act as smart cells or drug delivery systems for promoting endometrium regeneration.^[^ ^10^, ^31^ ^]^ For example, Chen et al. fabricated GelMA hydrogels carrying human umbilical cord mesenchymal stem cells (HUMSCs), which could release HUMSCs to facilitate endometrium regeneration and restore fertility.^[^ ^32^ ^]^ Therefore, in the current study, PLGA/GelMA fibers were used not only to improve biocompatibility but also to function as a smart drug delivery system to controllably and sustainably release E2 for modulating cell behavior and promoting uterine tissue regeneration.

**FIGURE 4:**
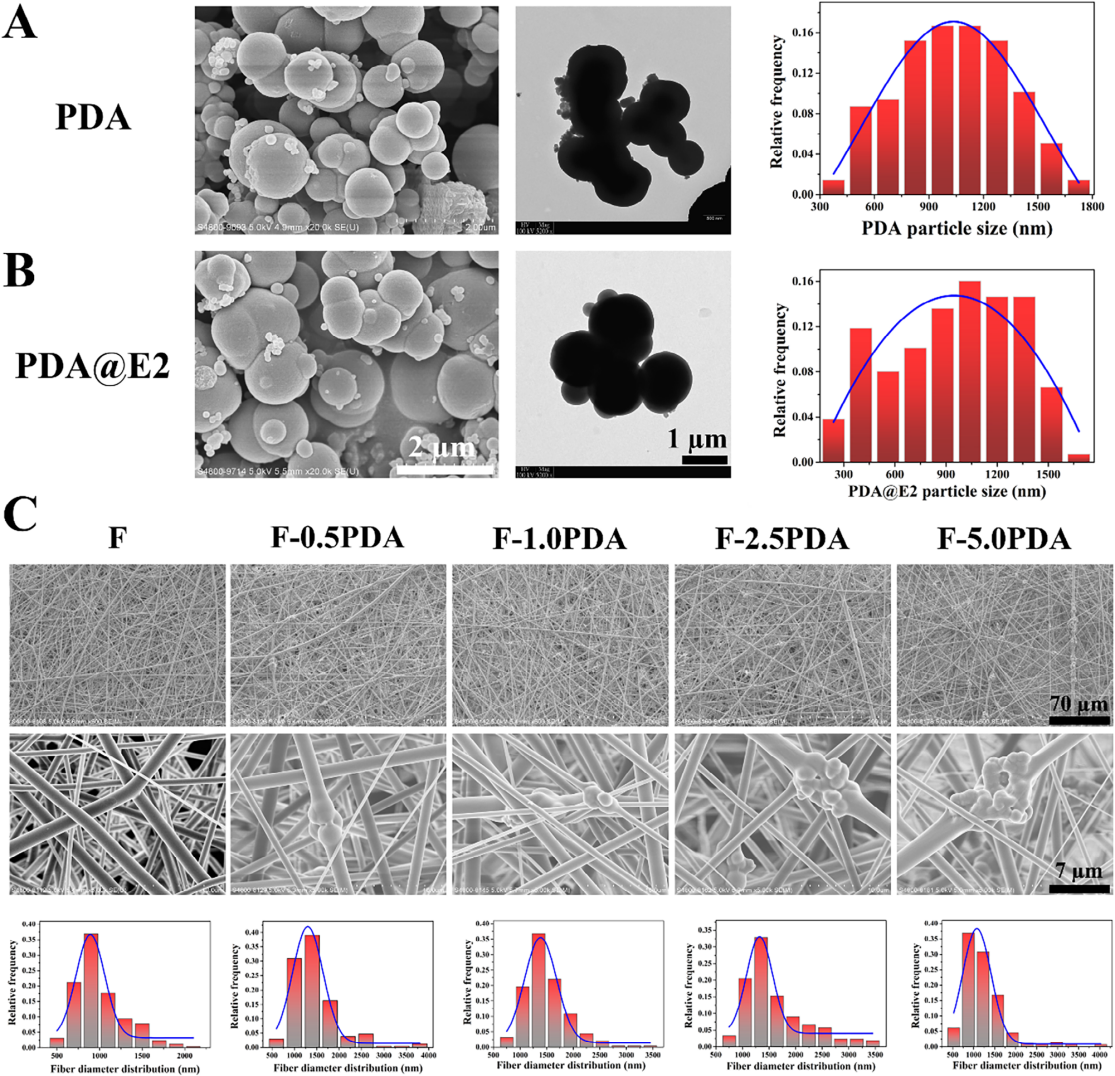
Particle morphology and size distribution of (A) PDA and (B) PDA@E2 particles. (C) Morphology and size distribution of electrospun PLGA/GelMA‐PDA fibers having different concentrations of PDA particles.

As evidenced by the ^1^H‐NMR and FTIR results shown in Figure S5, GelMA was successfully synthesized. PLGA and GelMA were then dissolved in HFIP solvent to prepare electrospinning solutions. Many factors, such as solution concentration, applied voltage and feeding rate can significantly affect the morphology, microstructure and diameter of the resulting electrospun fibers.^[^ ^33^ ^]^ To determine the optimal electrospinning parameters for PLGA/GelMA fibers, different applied voltages and feeding rates were used. It was observed that the average diameter of electrospun PLGA/GelMA fibers increased with the increase in feeding rate and with the decrease in applied voltage (Figures S6–S8). In the current study, the electrospun PLGA/GelMA fibers on PTMC/TPU scaffolds could significantly improve surface wettability and biocompatibility (Figure S9). Also, PLGA/GelMA fibers could work as an E2 loading and delivering system to controllably and sustainably release E2. Directly incorporating E2 in PLGA/GelMA electrospinning solution could impair E2 bioactivity, and the PLGA/GelMA fibers thus made may not provide controlled and sustained release of E2. Previous studies indicated that biomolecules directly incorporated in electrospun fibers had an initial burst release and exhibited low efficiency for long‐term therapeutic effects.^[^ ^34^ ^]^ Therefore, PDA@E2 particles were synthesized in the current study for delivering E2 in the designed and desired manner. PDA particles could work as an E2 delivery vehicle, protect its bioactivity and maintain its effectiveness. PDA particles have been popularly functioned as a drug delivery system because of their excellent biocompatibility, pH‐sensitive properties and photo‐thermal effect.^[^ ^5^, ^35^ ^]^ Because E2 is poorly water‐soluble, ethanol/water mixture was used to increase the solubility of E2 in the current study. The incorporation of E2 in PDA particles had little effect on PDA particle morphology and diameter. PDA particles had an average diameter of about 1,035 ± 15 nm, while the diameter of PDA@E2 particles was around 947 ± 50 nm (Figure 4A,B and Figure S10A). In UV–vis spectra, E2 had a characteristic absorption peak at 261 nm, while the absorption peak of PDA@E2 particles had a blue‐shift to 256 nm (Figure S10B).

Different concentrations of PDA particles were mixed with the PLGA/GelMA solution to construct electrospun fibers on PTMC/TPU scaffolds. As shown in Figure 4C, high concentration of PDA particles (5.0%) could cause particle aggregations in PLGA/GelMA‐PDA fibers, which would dramatically increase fiber diameter (Figure S11) and affect mechanical properties.^[^ ^36^ ^]^ Canales et al. showed that the diameter and morphology of electrospun poly(lactic acid) (PLA) fibers were significantly influenced by the incorporation of high bioglass particle concentration.^[^ ^37^ ^]^ They pointed out that due to the formation of bioglass particle aggregates, the mechanical strength of electrospun fibers encapsulated with 20% bioglass particles decreased from 0.2 to 0.04 MPa. In the current study, the PDA encapsulation in PLGA/GelMA fibers increased the tensile strength but decreased the elongation of the fibers. When the concentration of PDA particles was at 5%, PLGA/GelMA‐PDA fibers had the lowest elongation at fracture (around 60%). When the PDA particle concentration was at 2.5%, PLGA/GelMA‐PDA fibers had the highest tensile strength (about 4.5 MPa), but their elongation at fracture (about 100%) did not dramatically decrease. Based on these results, electrospun PLGA/GelMA fibers having 2.5% PDA or PDA@E2 particles were chosen for subsequent experiments.

**FIGURE 5:**
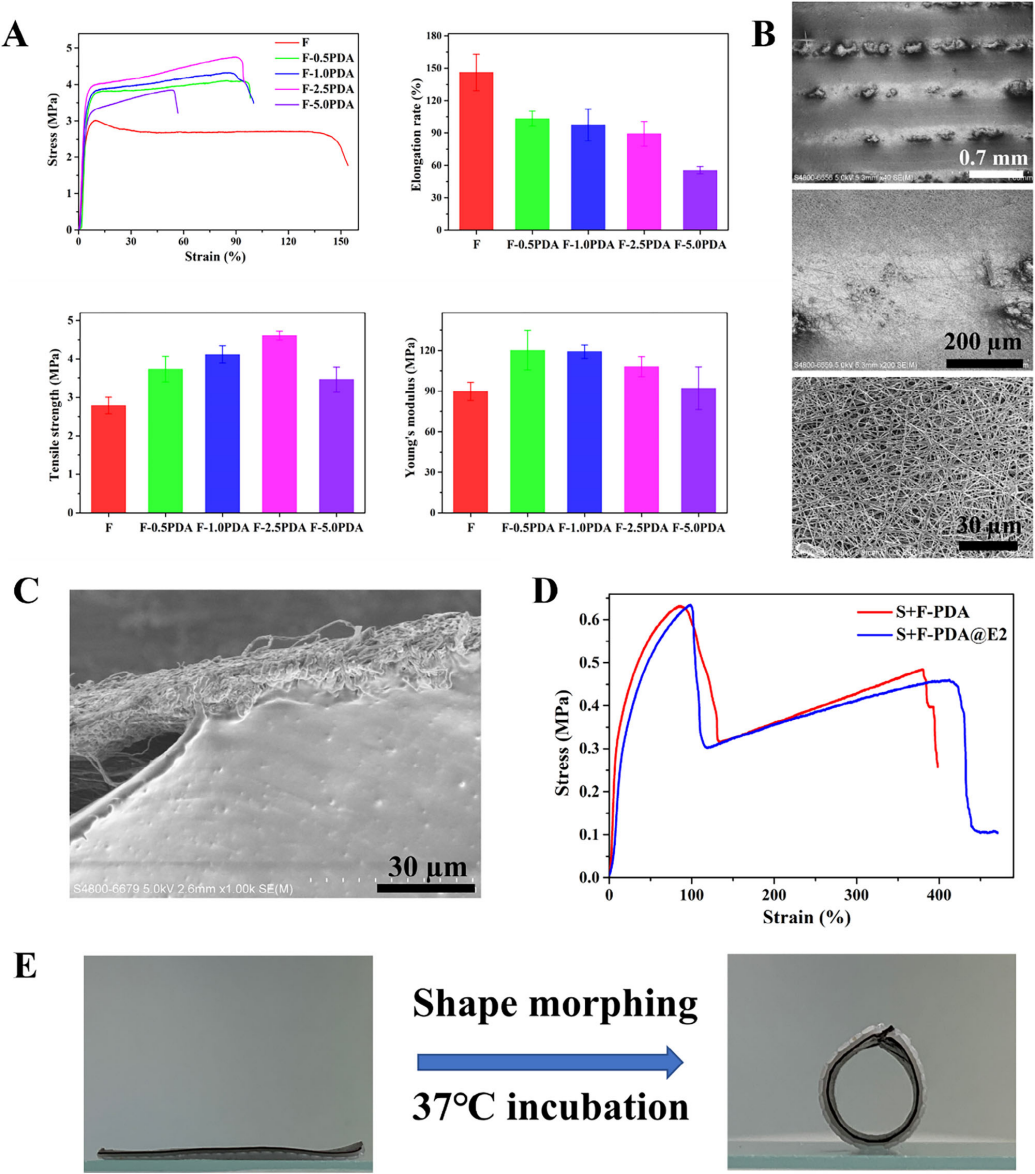
(A) Mechanical behavior and properties of electrospun PLGA/GelMA‐PDA fibers. (B) SEM images showing the surface morphology of a bilayer S+F‐PDA scaffold. (C) SEM image showing the cross‐sectional view of a bilayer S+F‐PDA scaffold. (D) Characteristic tensile stress‐strain curves of bilayer S+F‐PDA scaffolds and S+F‐PDA@E2 scaffolds. (E) Shape morphing of a bilayer S+F‐PDA scaffold from its planar shape to a tubular structure when immersed in the culture medium at 37°C. (The black layer was the PLGA/GelMA‐PDA@E2 fibrous layer.).

The bilayer scaffolds were constructed by fabricating PLGA/GelMA‐PDA fibers on PTMC/TPU scaffolds (Figure 1B). In previous studies by others, there were problems with the integration of two distinct layers for fabricating bilayer scaffolds.^[^ ^38^ ^]^ To tackle the interface integration problem, after fabricating PLGA/GelMA‐PDA fibers on PTMC/TPU scaffolds, the bilayer scaffolds were incubated in an oven at 50°C overnight. Since PTMC/TPU scaffolds had low *T*_g_ temperatures (Figure 2C), the glass transition of PLGA took place at 33°C and PLGA became very viscous at about 50°C (Figure S12). The high temperature incubation would make PLGA/GelMA‐PDA fibers and PTMC/TPU scaffolds attached to each other. SEM images in Figure 5B,C show the surface and cross‐sectional views of bilayer scaffolds. It could be seen that electrospun fibers fully covered the PTMC/TPU scaffold surface. The cross‐sectional view showed that fibers were closely attached to the PTMC/TPU scaffold surface. In addition, as shown in Figure S13, at the initial stage of electrospinning, PLGA/GelMA‐PDA fibers merged with the PTMC/TPU scaffold surface. Subsequently, the mechanical properties of bilayer scaffolds were investigated using tensile tests. The tensile strain‐stress curve in Figure 5D showed that the mechanical behavior of bilayer scaffolds could be divided into two phases: (1) the break of electrospun fiber and (2) the break of PTMC/TPU scaffolds. The tensile strain and strength of S+F‐PDA and S+F‐PDA@E2 bilayer scaffolds (Table S2) were 315.54 ± 57.47 % and 337.44 ± 38.96%, and 0.6 ± 0.06 and 0.61 ± 0.10 MPa, respectively, which are superior to natural uterine tissues. The shape morphing behavior of bilayer scaffolds was also investigated. Video S2 recorded the shape morphing process of the S+F‐PDA@E2 bilayer scaffold, showing that electrospun fibers had little effect on the shape morphing ability of bilayer scaffolds. The bilayer scaffolds could easily return to their permanent tubular shape when immersed in the culture medium at 37°C (Figure 5E).

### 3.3 Photothermal effect and E2 release from bilayer scaffolds

**FIGURE 6:**
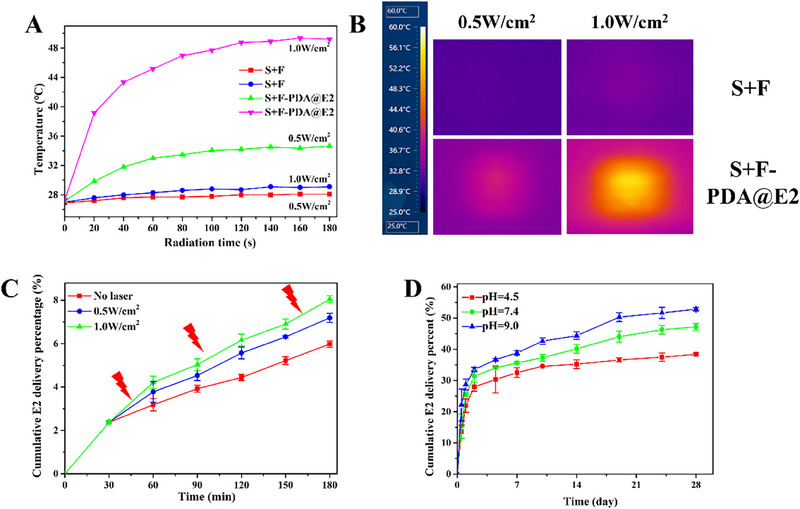
Photothermal effect and E2 release behavior of bilayer scaffolds. (A) Temperature changes of S+F and S+F‐PDA@E2 bilayer scaffolds under the irradiation of an 808 nm NIR laser at different energy densities (0.5 and 1.0 W cm^−2^). (B) Infrared thermal images of S+F and S+F‐PDA@E2 bilayer scaffolds after a 3‐min NIR laser irradiation. (C) In vitro E2 cumulative release curves of S+F‐PDA@E2 bilayer scaffolds with (0.5 and 1.0 W cm^−2^, respectively) or without NIR irradiation (pH 7.4). (D) In vitro E2 cumulative release curves of S+F‐PDA@E2 bilayer scaffolds in pH 4.5, 7.4, and 9.0 solutions, respectively.

The application of PDA particles in smart drug delivery systems for controlled and sustained release of biomolecules has been considered an efficient way to promote tissue regeneration.^[^ ^39^ ^]^ Previous studies have already indicated that oral administration of E2 would have a very poor therapeutic effect in treating uterine injuries as compared to in situ controlled release.^[^ ^40^ ^]^ PDA is an excellent photothermal agent with good biocompatibility and biodegradability. Moreover, due to their inherent molecule structure, PDA particles exhibit pH‐sensitive behavior. Therefore, the release of biomolecules from PDA particles can be easily regulated by NIR laser irradiation and environment pH. In the current study, since E2 as a biomolecule for uterine regeneration was encapsulated in PDA particles and the synthesized PDA@E2 particles were incorporated in electrospun fibers, the release of E2 from S+F‐PDA@E2 bilayer scaffolds could be precisely tuned by NIR laser irradiation and/or environmental pH. Firstly, the photothermal effects of S+F and S+F‐PDA@E2 bilayer scaffolds were investigated. Figure 6A,B display temperature changes of S+F and S+F‐PDA@E2 bilayer scaffolds under the irradiation of the NIR laser at the energy densities of 0.5 and 1.0 W cm^−2^, respectively. S+F‐PDA@E2 bilayer scaffolds could be heated up to 35 and 49°C after being irradiated by 0.5 and 1.0 W cm^−2^ NIR lasers, respectively, for 3 min. Such high temperatures caused by the NIR laser could accelerate the Brownian movements of E2 molecules. The accelerated movement of E2 molecules would further result in more E2 being released from bilayer scaffolds. Indeed, with the irradiation of the NIR laser, more E2 was released in comparison to unirradiated samples (Figure 6C).

On the other hand, because the human uterus presents a dynamic pH environment, it is essential to study E2 release kinetics in buffer solutions of different pH values. In the current study, the E2 release behavior was studied in solutions of pH 4.5, 7.4, and 9.0, respectively. As shown in Figure 6D, E2 could be sustainably released in the long‐term (over 28 days), and the E2 release kinetics could match the Higuchi model (Figure S14). The sustained release of E2 would have a significant therapeutic effect on uterine regeneration. It is well‐known that long‐term biomolecule delivery can prolong the beneficial effect and thus promote tissue regeneration.^[^ ^41^ ^]^ Moreover, in contrast to an acidic environment, E2 could be quickly released in a basic solution, which may be attributed to the disintegration of PDA particles and degradation of PLGA/GelMA electrospun fibers in an alkaline environment.

Estrogen plays a critical role in regulating menstruation and endometrium regeneration. The safety and efficacy of E2 for endometrium regeneration have been clinically proven.^[^ ^42^ ^]^ Endometrium stromal cells and glandular epithelial cells in the endometrium have many estrogen receptors. When E2 binds to their receptors, it enables increases in the expressions of angiogenic growth factors such as VEGF, bFGF and TGF‐β1.^[^ ^43^ ^]^ These angiogenic growth factors would facilitate endothelial cell migration, proliferation, differentiation, tube formation, and thus increase angiogenesis and blood vessel density, thereby improving the reconstruction of uterine tissues. Previous studies have investigated the possibility in using hydrogels or scaffolds to deliver E2 in situ for uterus injury treatment.^[^ ^40^, ^44^ ^]^ However, the problem was that E2 released from these hydrogels or scaffolds could not reach a controlled and sustained long‐term delivery state. In the current study, the in‐site release of E2 from bilayer scaffolds could not only be regulated by the NIR laser irradiation but also modulated by environment pH, which is highly beneficial for uterine regeneration.

### 3.4 In vitro biological performance of bilayer scaffolds

**FIGURE 7:**
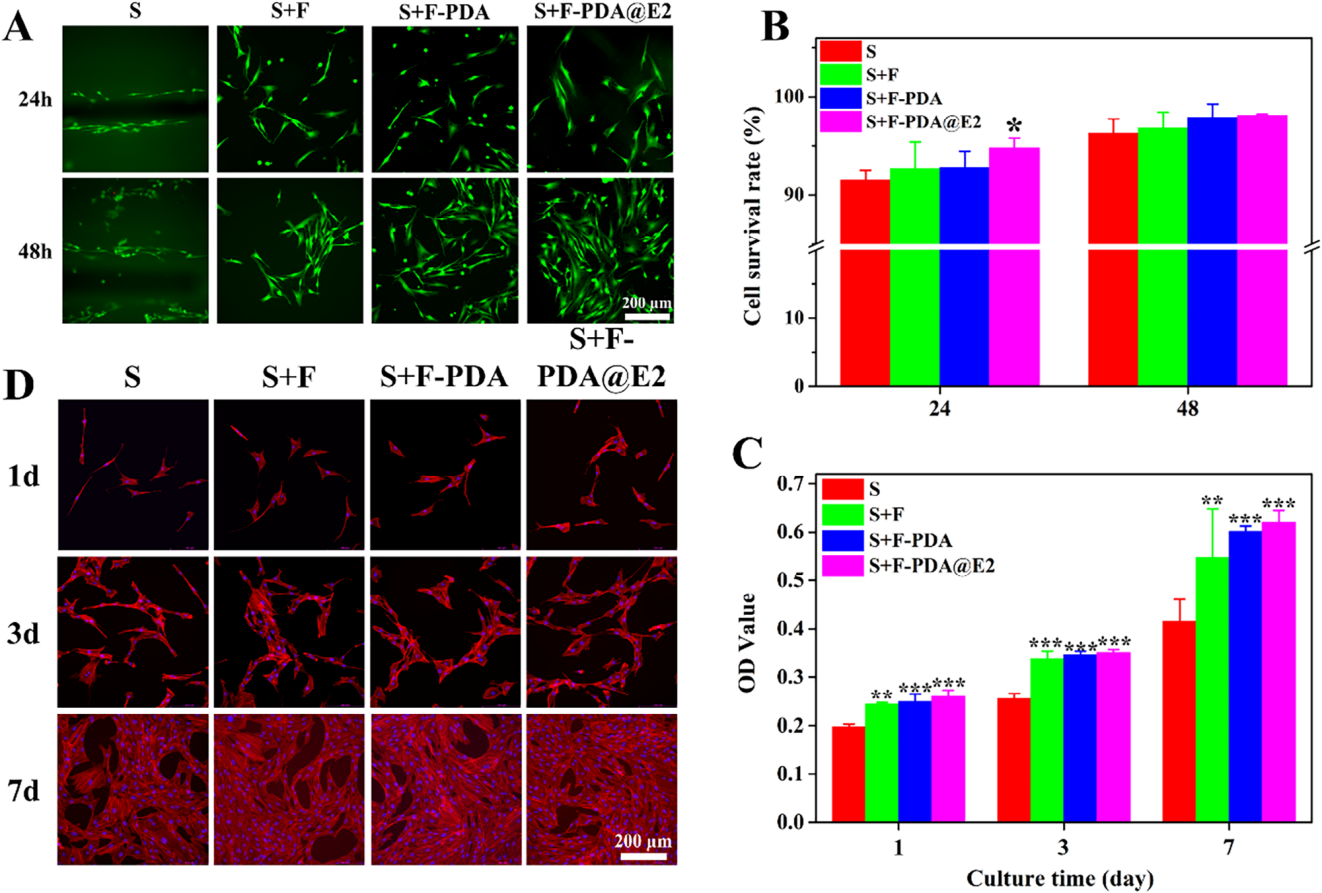
In vitro biological performance of S+F‐PDA@E2 scaffolds. (A) Live/dead fluorescence images for BMSCs cultured on bilayer scaffolds. (B) Cell viability of BMSCs on bilayer scaffolds after culture for 24 and 48 h, respectively. (C) Proliferation of BMSCs on bilayer scaffolds. (D) F‐actin and nucleus fluorescence images of BMSCs on bilayer scaffolds after culture for 1, 3 and 7 days, respectively.

Many in vitro and in vivo studies have demonstrated the safety and efficacy of menstrual‐derived stem cells (MenSCs) obtained from women's menstrual fluids for repairing damaged uterine tissues.^[^ ^45^ ^]^ Although MenSCs attract growing interest in clinical applications because they are multipotent and has high proliferation rate, the limited source and complex extraction procedure are problematic. A recent study has indicated that BMSCs had comparable properties as MenSCs,^[^ ^46^ ^]^ suggesting that BMSCs could replace MenSCs for use in uterine regeneration. Additionally, many studies have demonstrated the efficacy of BMSCs for uterine regeneration in preclinical and clinical trials. BMSCs could differentiate into endometrium epithelial cells so as to improve gland and blood vessel formation and activate the resident endometrium stem cells to promote uterine regeneration via the paracrine effect.^[^ ^10^, ^47^ ^]^ Therefore, in the current study, BMSCs were used to evaluate the in vitro biological performance of bilayer scaffolds. Cell attachment results, as shown in Figure S15, revealed that BMSCs after being cultured on S, S+F, S+F‐PDA and S+F‐PDA@E2 scaffolds for 1 day all exhibited spread morphology, suggesting that all scaffolds were biocompatible. The live/dead assay results indicated that the BMSC survival rate in S, S+F, S+F‐PDA and S+F‐PDA@E2 scaffolds was over 90% after cultured for 24 and 48 h, respectively (Figure 7A,B). However, the proliferation rates of BMSCs on those scaffolds were significantly different (Figure 7C). Compared to S scaffolds (i.e., PTMC/TPU), S+F bilayer scaffolds and S+F‐PDA and S+F‐PDA@E2 bilayer scaffolds had higher proliferation rates, suggesting that electrospun PLGA/GelMA fibers on PTMC/TPU scaffolds had made scaffold surface hydrophilic, improved scaffold‐cell interface behavior and thus promoted cell proliferation. Moreover, due to the PDA particles and sustained release of E2, S+F‐PDA@E2 bilayer scaffolds had the highest proliferation rate, which was consistent with previous studies.^[^ ^40^, ^44^ ^]^ Furthermore, phalloidin/DAPI staining results in Figure 7D indicated that BMSCs maintained their phenotypes and showed spindle‐like morphology on S+F, S+F‐PDA and S+F‐PDA@E2 bilayer scaffolds. Overall, S+F‐PDA@E2 bilayer scaffolds had excellent biocompatibility and promoted BMSC growth.

### 3.5 Fabrication and performance of biomimicking trilayer tissue engineering scaffolds

S+F‐PDA@E2 bilayer scaffolds providing controlled and sustained release of E2 are beneficial for endometrium stem cell migration, growth, proliferation and differentiation and, hence, can promote uterine regeneration. Damaged uterine tissues could significantly affect the growth of endometrium stem cells, which are essential for the dynamic, cyclic processes of growth, differentiation, sloughing, and renewal of endometrium. Therefore, introducing autologous stem cells to the damaged site in the uterus could stimulate and activate endometrium stem cells to grow, proliferate, and differentiate.^[^ ^48^ ^]^ After implantation, autologous stem cells would secrete matrix proteins to build up extracellular matrix (ECM) and attract surrounding cells to migrate towards the damaged site via auto/paracrine effect, thereby facilitating tissue remodeling. Meanwhile, new tissue engineering strategies can combine scaffolds, stem cells and biomolecules for treating tissue damage. 3D bioprinting, a recently emerged technological cluster in additive manufacturing (AM), shows increasing applications in tissue engineering because of its ability to construct hydrogel‐based 3D structures with a high spatial distribution of cells.^[^ ^18^, ^49^ ^]^ Compared with conventional biomanufacturing technologies, 3D bioprinting provides high cell‐loading efficiency and more homogenous cell distribution within the constructs and can load and release cells to the damaged sites on‐target. Therefore, in the current study, to introduce abundant autologous stem cells at uterine tissue repair sites, BMSC‐laden hydrogels were 3D printed on bilayer scaffolds for achieving the designed ultimate trilayer scaffolds for uterine tissue regeneration.

**FIGURE 8:**
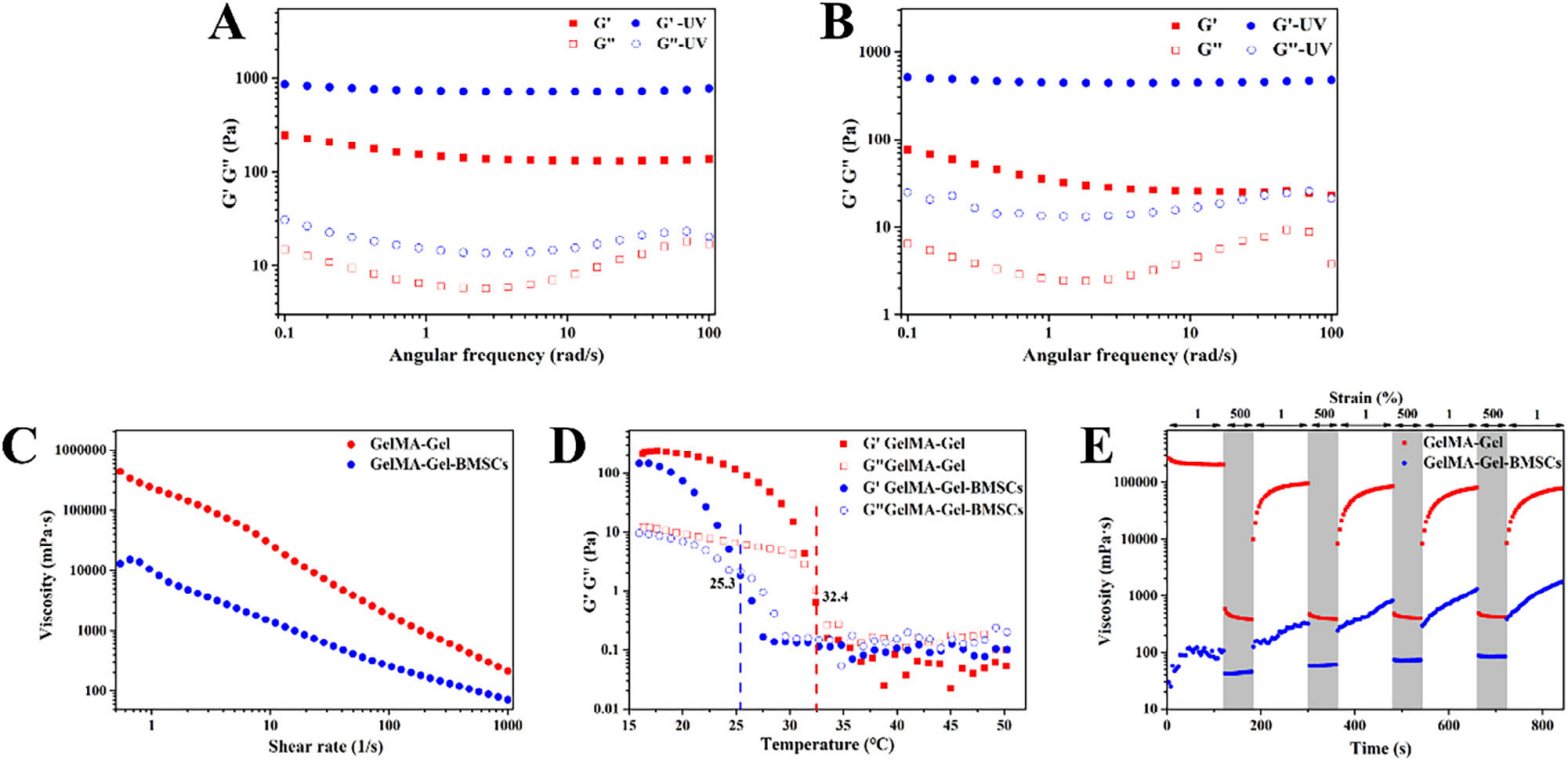
Rheological properties of GelMA/Gel inks and GelMA/Gel‐BMSC bioinks. (A) Storage modulus (*G*′) and loss modulus (*G*ʺ) of GelMA/Gel inks at 20°C with and without UV crosslinking. (B) *G*′ and *G*ʺ of GelMA/Gel‐BMSC bioinks at 20°C with and without UV crosslinking. (C) Shear thinning behavior of GelMA/Gel inks and GelMA/Gel‐BMSC bioinks. (D) Variation of *G*′ and *G*ʺ in terms of temperature for GelMA/Gel inks and GelMA/Gel‐BMSC bioinks. (E) Viscosity of GelMA/Gel inks and GelMA/Gel‐BMSC bioinks in thixotropic tests.

GelMA‐based bioinks have been widely used for 3D bioprinting due to their high biocompatibility, controllable biodegradation rate and photopolymerizable ability.^[^ ^50^ ^]^ So far, many studies have used GelMA‐based bioinks to carry different cells for repairing various damaged body tissues, including bone, articular cartilage, blood vessel and skin. The application of 3D bioprinted GelMA hydrogels loaded with human induced pluripotent stem cell‐derived mesenchymal stem cells (hiMSCs) for uterine endometrium regeneration was initially reported in 2020.^[^ ^10c^ ^]^ Ji et al. showed that hiMSC‐loaded GelMA‐based hydrogels could significantly increase the survival duration of incorporated hiMSCs and promote the recovery of the endometrial histomorphology and the regeneration of endometrial cells and endothelial cells. Therefore, in the current study, GelMA‐based bioinks loaded with BMSCs were prepared to fabricate a cell‐laden hydrogel layer on S+F‐PDA@E2 bilayer scaffolds, forming the designed biomimicking trilayer scaffolds. Since pure GelMA inks usually exhibit poor printability, GelMA/Gel inks were used. Figure 8 provides the rheological properties of GelMA/Gel inks and GelMA/Gel‐BMSC bioinks. Figure 8A,B show that the *G*′ and *G*ʺof GelMA/Gel‐BMSC bioinks dramatically decreased in comparison with GelMA/Gel inks owing to the addition of BMSCs. Moreover, because of the loading of BMSCs in GelMA/Gel bioinks, the shear‐thinning behavior became weak and the gel‐sol transition temperature decreased from 32.4 to 25.3°C (Figure 8C,D). Thixotropic tests were conducted to evaluate the recoverability of bioinks. Once bioink is extruded from the nozzle tip, it is relieved from the high shear stress in the nozzle and should have the ability to quickly recover to its initial state.^[^ ^51^ ^]^ Compared to GelMA/Gel inks, GelMA/Gel‐BMSC bioinks were less able to recover their initial state (Figure 8E). The loading of BMSCs in GelMA/Gel bioinks therefore weakened their rheological properties, which may be attributed to the interference of BMSCs. The introduced cells in polymer solutions should have interfered with the interaction between polymer chains.

**FIGURE 9:**
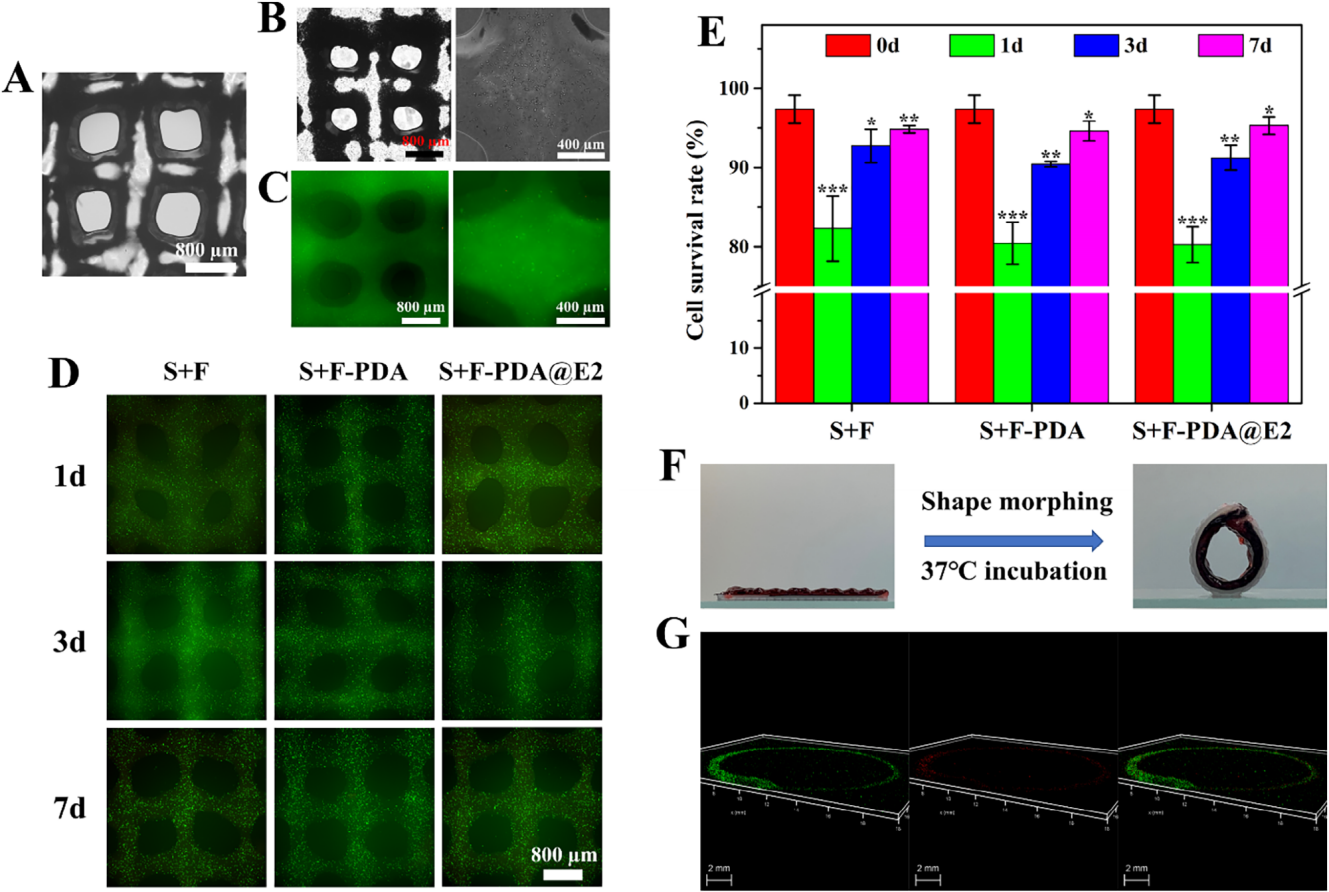
Characteristic of biomimicking trilayer scaffolds containing a 3D bioprinted BMSC‐laden hydrogel layer. (A) An optical image of 3D printed GelMA/Gel hydrogel. (B) Optical images and (C) fluorescence images of 3D bioprinted BMSC‐laden hydrogel. (D) Live/dead assay results of BMSCs in trilayer scaffolds after culturing for 1, 3 and 7 days. (E) Cell survival rates of BMSCs in trilayer scaffolds after culturing for 1, 3 and 7 days. (F) Shape morphing of a trilayer scaffold from the planar shape to tubular structure when cultured at 37°C. (The red layer in the scaffold was the 3D bioprinted BMSC‐laden hydrogel layer.) (G) CLSM images showing the 3D distribution of BMSCs in trilayer scaffolds after scaffold shape morphing at 37°C.

After the rheological studies, GelMA/Gel‐BMSC bioinks were 3D printed on S+F‐PDA@E2 bilayer scaffolds. As shown in Figure 9A,B, BMSCs were homogeneously distributed in the printed hydrogel layer after 3D bioprinting. However, due to the incorporation of BMSCs, GelMA/Gel‐BMSC hydrogels exhibited lower printability than GelMA/Gel hydrogels (Figure S16). Previously, Schwartz et al. investigated the effect of cell encapsulation on the printability of bioinks.^[^ ^52^ ^]^ They found that cell encapsulation in gelatin bioinks impaired 3D bioprinting resolution and that a high cell density could significantly affect the printability of gelatin bioinks. As discussed above, this phenomenon could have resulted from the loose connections among neighboring polymer chains, as the cells may have blocked their direct contact, causing the reduction in bioink viscosity. In the cell survival study, the live/dead assay results shown in Figure 9C indicated that BMSCs had very high cell survival rates in the hydrogels after 3D bioprining, suggesting that the shear stress in the 3D bioprinting process had little effect on cell apoptosis. Moreover, the cell survival rate of BMSCs dramatically decreased to ≈82% after cultured for 1 day but recovered to ≈95% after cultured for 7 days (Figure 9D,E). The structure of BMSC‐laden hydrogels started to disintegrate as the culture time increased (Figure 9D). Also, the cell survival rates of BMSCs in the 3D printed hydrogel layer on S+F, S+F‐PDA and S+F‐PDA@E2 layered scaffolds did not show a significant difference.

The uterus has a hierarchical and curved structure. Although a basic trilayer tissue engineering scaffold can have a multilayered biomimicking structure and comparable mechanical strength with natural uterus, the planar, static shape of a trilayer scaffold is not sufficient to meet the implantation requirement. Shape memory polymers with the ability to change shapes of their products upon suitable stimuli have distinct advantages for obtaining curved or tubular scaffolds.^[^ ^53^ ^]^ Owing to programmed shape morphing of the PTMC/TPU scaffold layer, the trilayer scaffolds produced in the current study could transform from the planar shape to tubular structures when cultured at 37°C, as shown in Figure 9F and Video S3. To better visualize the trilayer structure of the scaffolds, a red dye was added in the hydrogel layer for scaffolds in the control group. The trilayer structure could therefore be seen clearly in the tissue engineering scaffolds (Figure 9F): the white outlayer was a 3D printed PTMC/TPU scaffold layer with high elasticity, the black interlayer was electrospun PLGA/GelMA fibers incorporated PDA@E2 particles and the red innerlayer was 3D bioprinted BMSC‐laden GelMA/Gel hydrogel. Furthermore, the CLSM images in Figure 9G revealed that BMSCs were homogeneously distributed in the inner GelMA/Gel hydrogel layer of trilayer scaffolds. As a result, compared to scaffolds made and investigated by other researchers, the trilayer scaffolds fabricated in this study had: (1) comparable mechanical strength with the native uterus, (2) a trilayer structure that mimicked the hierarchical structure of the uterus, (3) controlled and sustained release of E2 to regulate cell behavior, and (4) shape morphing ability to form curved or tubular structure after implantation. Such trilayer scaffolds have a high potential for uterine regeneration applications.

## 4 CONCLUSIONS

In the current study, a trilayer tissue engineering scaffold mimicking the hierarchical structure of native uterine tissue was designed and trilayer scaffolds of this design were successfully constructed through 4D printing, electrospinning and 3D bioprinting. The trilayer scaffolds had three distinct layers: 4D printed PTMC/TPU scaffold outlayer with shape morphing ability and high stretchability, electrospun PLGA/GelMA‐PDA@E2 fibrous interlayer with photothermal effect and controlled and sustained E2 release, and 3D bioprinted BMSC‐laden hydrogel innerlayer providing abundant stem cells at the repair/tissue regeneration site. These trilayer scaffolds were highly stretchable and exhibited comparable mechanical properties with native uterine tissue. Additionally, they could deliver E2 controllably and sustainably. The E2 release from trilayer scaffolds was also pH‐responsive and could be tuned by NIR laser irradiation. PLGA/GelMA‐PDA@E2 fibers fabricated on the PTMC/TPU scaffold made the scaffold surface more hydrophilic and improved biological performance. BMSCs encapsulated in GelMA/Gel bioinks exhibited high survival rates in the 3D bioprinted hydrogel layer of the trilayer scaffolds. Importantly, the biomimicking trilayer scaffolds had shape morphing ability, transforming from a planar shape to curved or tubular structures when immersed in the culture medium at 37°C. The biomimicking trilayer tissue engineering scaffolds designed and fabricated in the current study have a high potential for uterine tissue regeneration.

## AUTHOR CONTRIBUTIONS

**Shangsi Chen**: Design of the research; methodology; investigation; writing; revision. **Junzhi Li**: Methodology; investigation; revision. **Liwu Zheng**: Reviewing; editing. **Jie Huang**: Reviewing; editing. **Min Wang**: Design of the research; research supervision; writing; reviewing; revision and editing.

## CONFLICT OF INTEREST STATEMENT

The authors declare no conflicts of interest.

## Supporting information

Supporting Information

Supporting Information

Supporting Information

Supporting Information

## Data Availability

The data that support the findings of this study are available from the corresponding author upon reasonable request.
