# Supplementary material for: Biomimicking trilayer scaffolds with controlled estradiol release for uterine tissue regeneration
Source: Exploration (Beijing). 2024 Apr 17;4(5):20230141. doi: 10.1002/EXP.20230141 (PMC11491300; doi:10.1002/EXP.20230141)
Supplement: Supplementary file 1 — Supporting Information [file EXP2-4-20230141-s002.docx]

*Supporting Information*

**Biomimicking Trilayer Scaffolds with Controlled Estradiol Release for Uterine Tissue Regeneration**

Shangsi Chen ^a^, Junzhi Li ^a^, Liwu Zheng ^b^, Jie Huang ^c^, Min Wang ^a, *^

^a^ Department of Mechanical Engineering

The University of Hong Kong

Pokfulam Road, Hong Kong

^b^ Faculty of Dentistry

The University of Hong Kong

34 Hospital Road, Sai Ying Pun, Hong Kong

^c^ Department of Mechanical Engineering

University College London

Torrington Place, London WC1E 7JE, U.K.

**Keywords:** 4D printing, 3D bioprinting, electrospinning, controlled release, shape morphing

* Corresponding Author:

Professor Min Wang, at The University of Hong Kong, Hong Kong

Email: memwang@hku.hk Tel: +852 3917 7903 Fax: +852 2858 5415


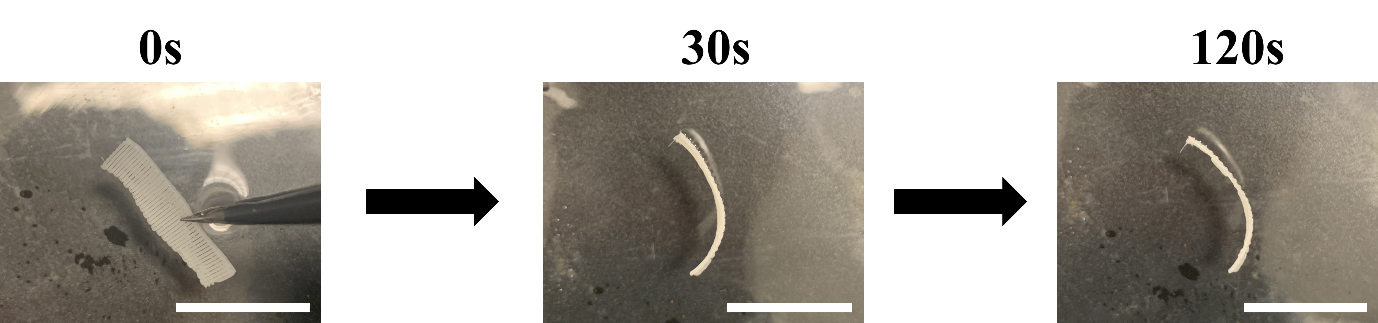


Figure S1 Representative images of a PTMC/TPU scaffold at the PTMC:TPU ratio of 0.125:1 after immersion in the culture medium at 37 ℃ for different times. (Scale bar: 20 mm.)


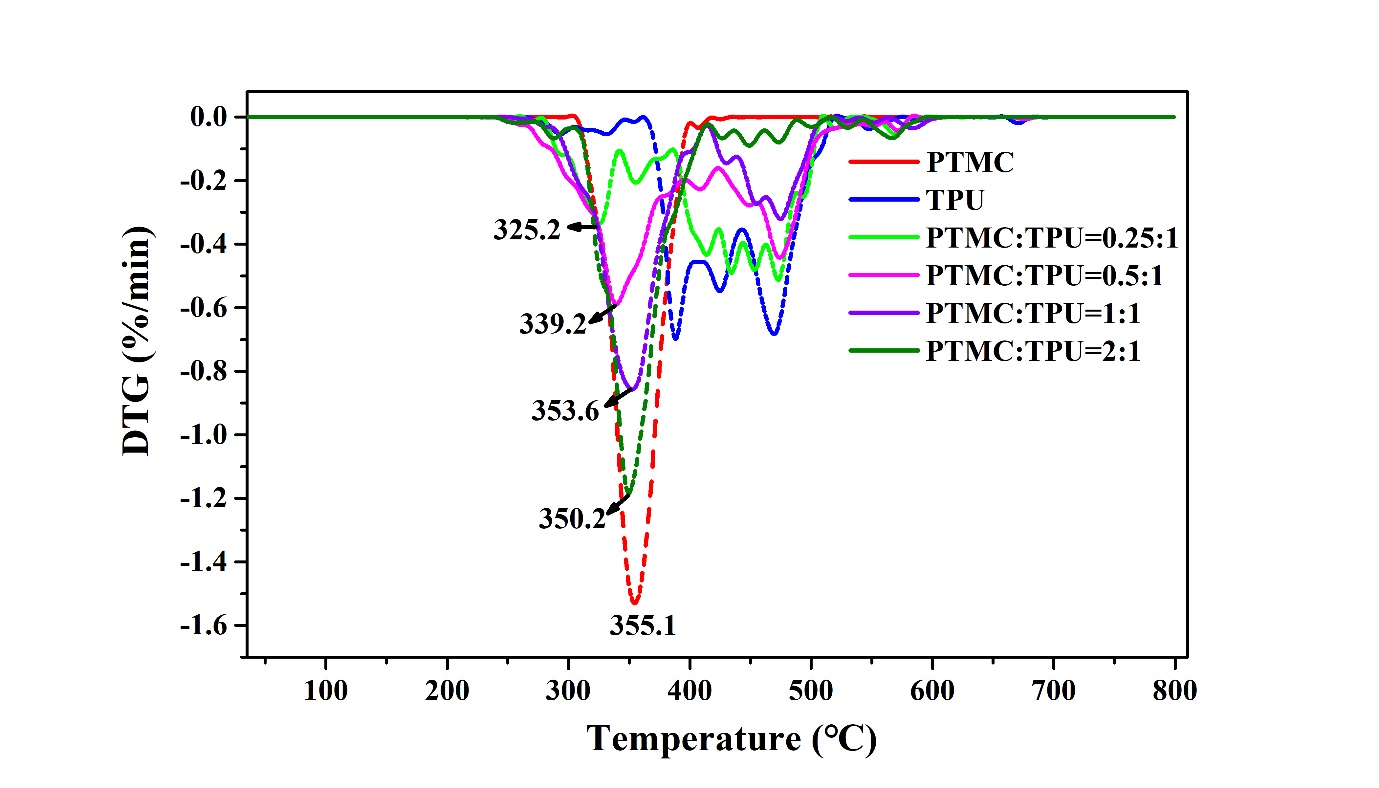


Figure S2 DTG curves of 3D printed PTMC, TPU and PTMC/TPU scaffolds.


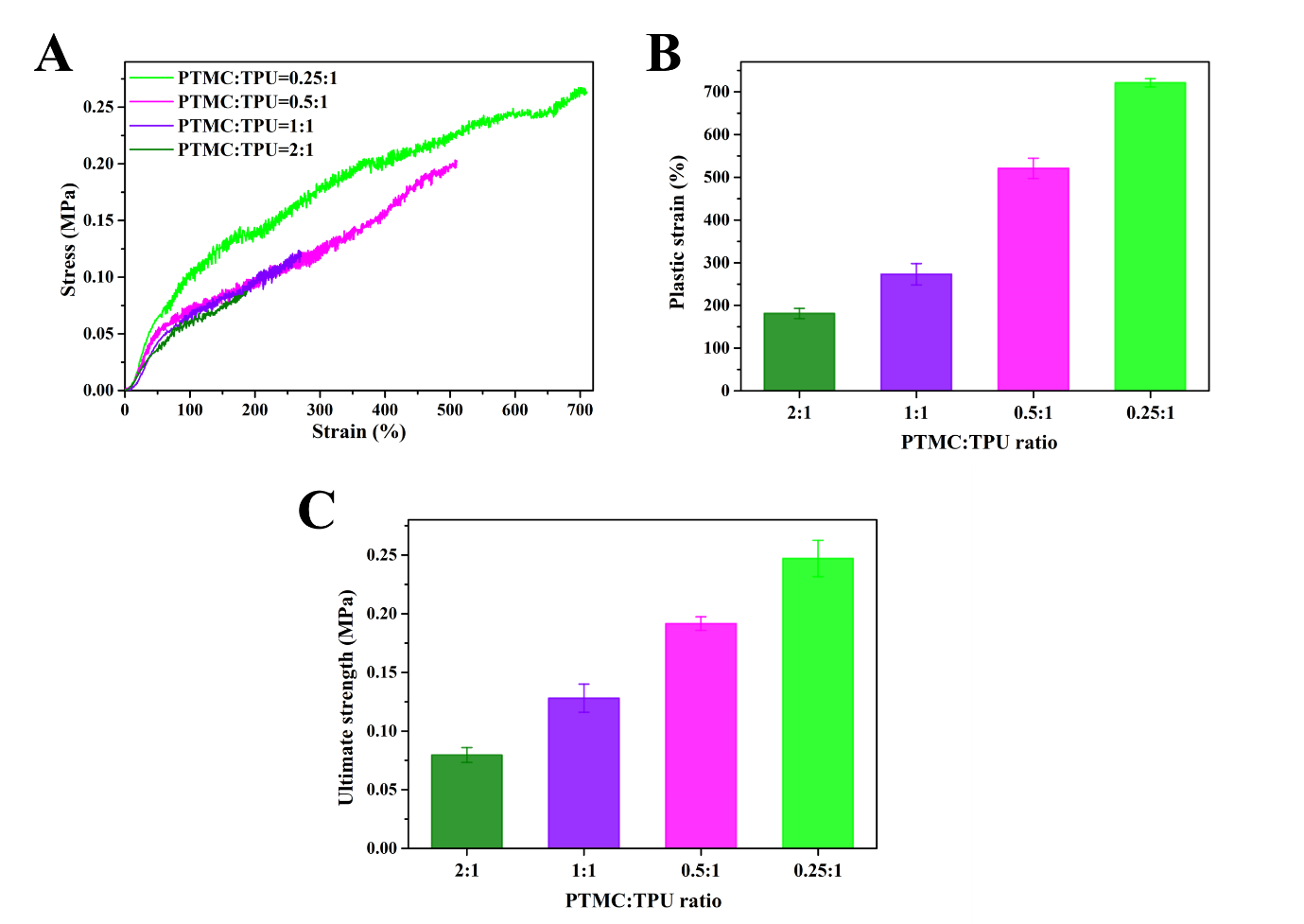


Figure S3 (A) Tensile stress-strain curves, (B) plastic strain and (C) ultimate tensile strength of PTMC/TPU scaffolds tested at 37 ℃.


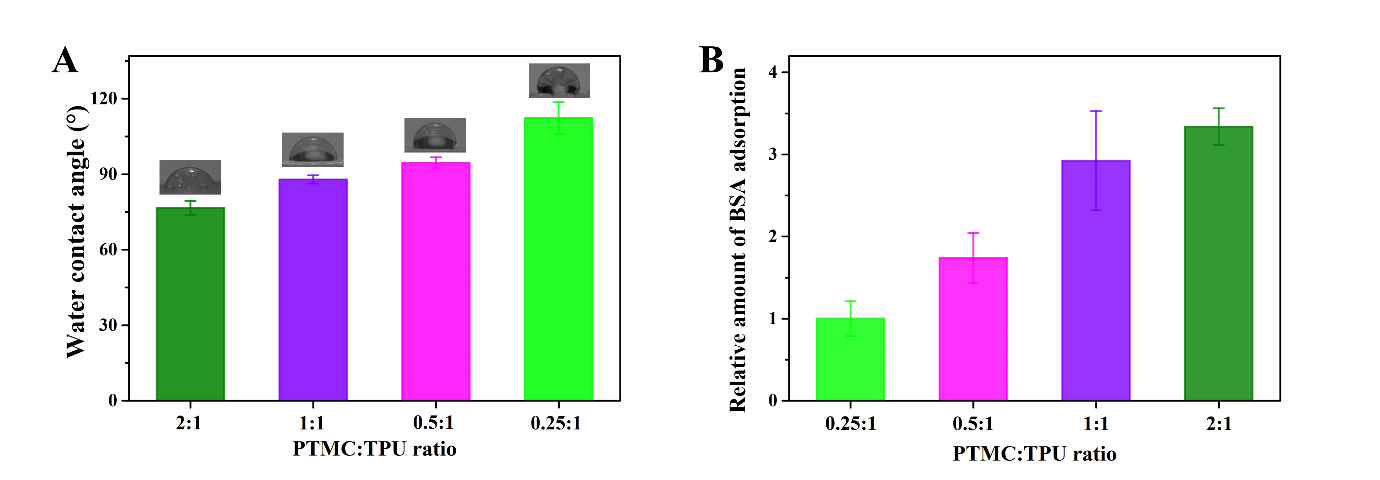


Figure S4 (A) Water contact angle and (B) relative amount of BSA adsorption by PTMC/TPU scaffolds of different PTMC:TPU ratios.


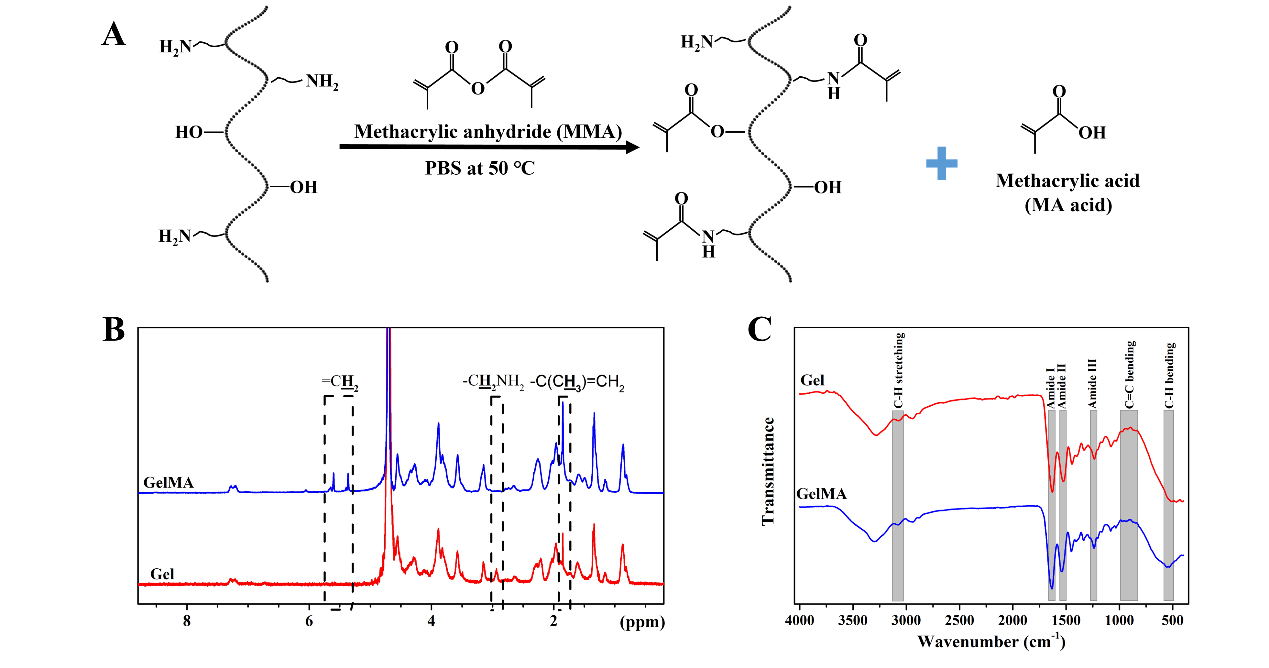


Figure S5 (A) Schematic illustration of GelMA synthesis. (B) ^1^H-NMR and (C) FTIR spectra of Gel and GelMA.


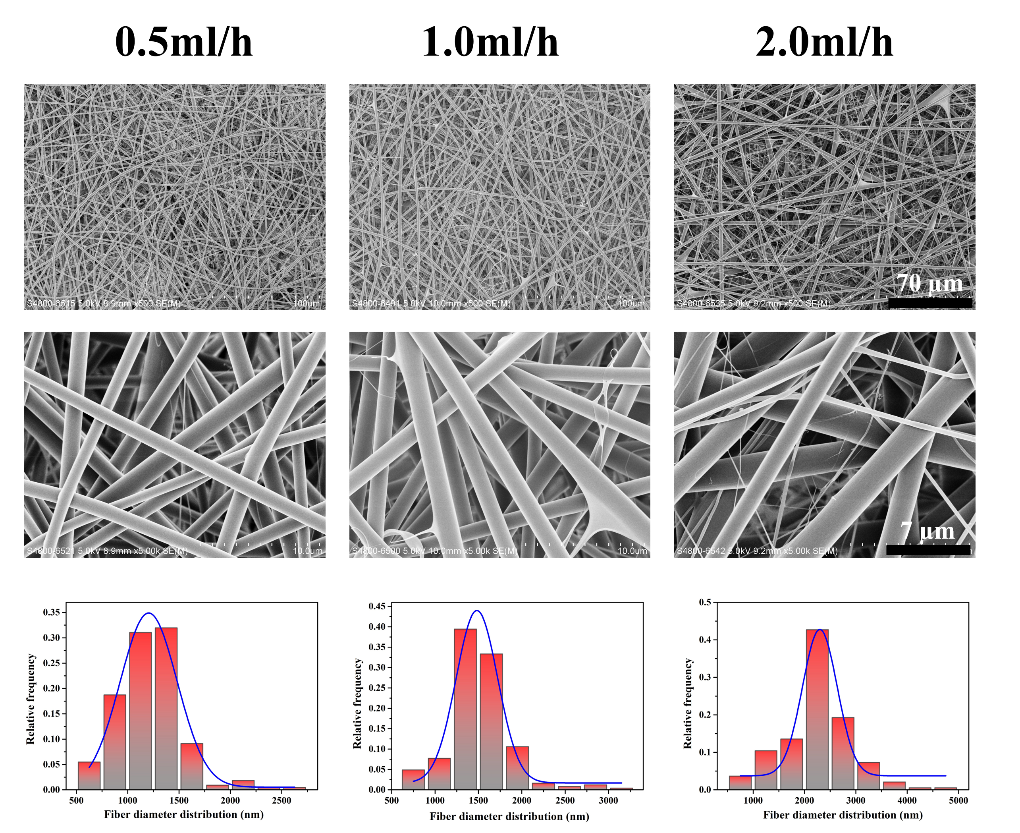


Figure S6 SEM images and diameter distribution of electrospun PLGA/GelMA fibers at different feeding rates (Applied voltage: 10 kV).


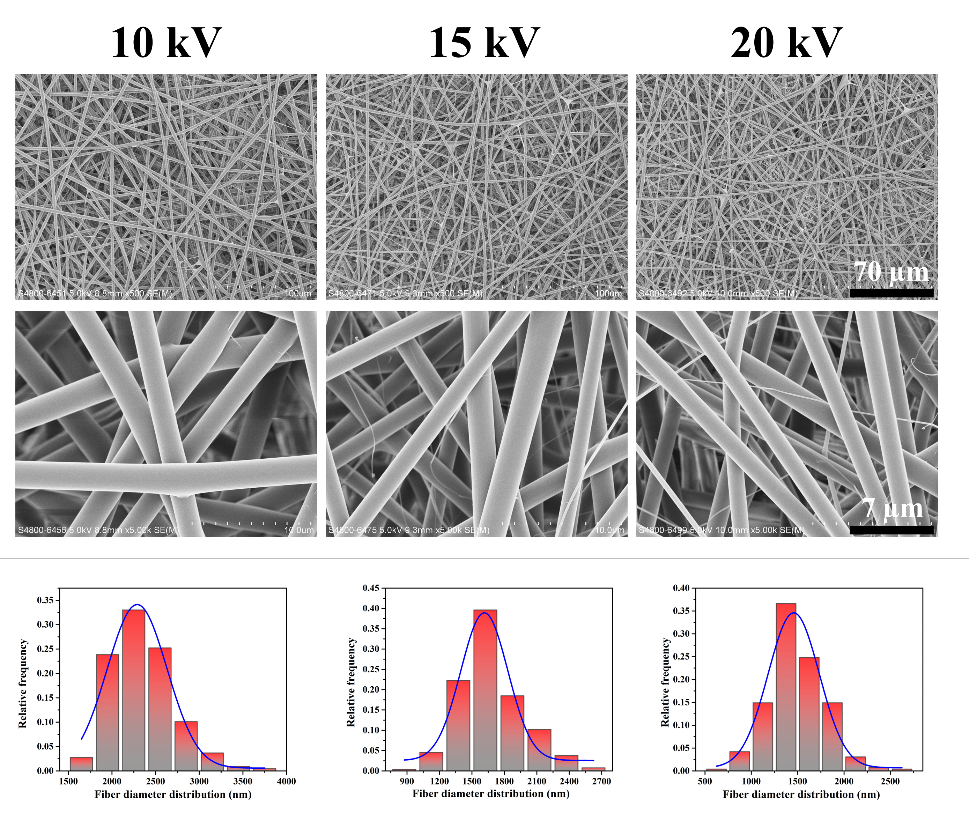


Figure S7 SEM images and diameter distribution of electrospun PLGA/GelMA fibers at different applied voltages (Feeding rate: 2.0 ml h^-1^).


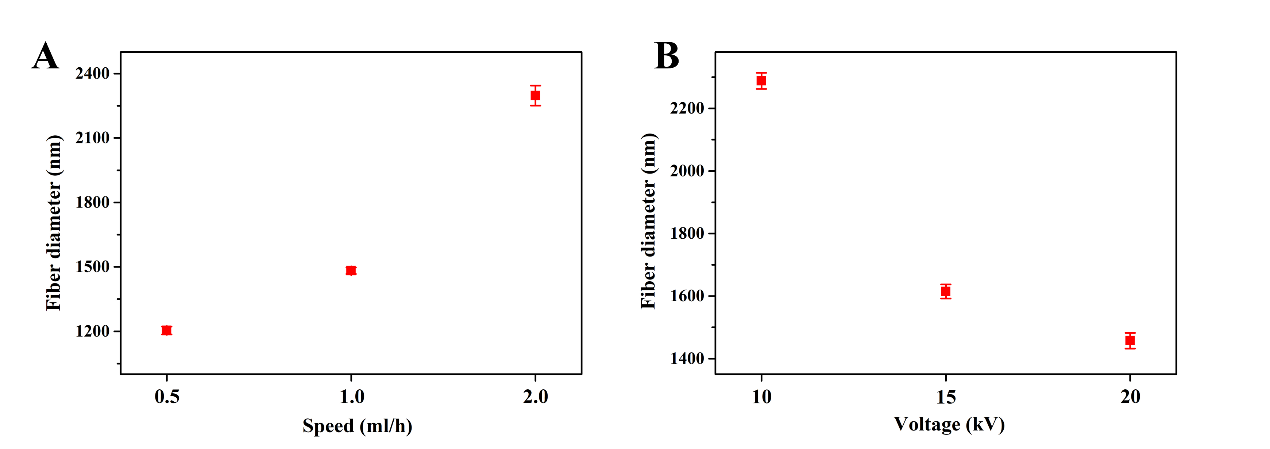


Figure S8 Fiber diameter analysis for electrospun PLGA/GelMA fibers at different (A) feeding rates and (B) applied voltages.


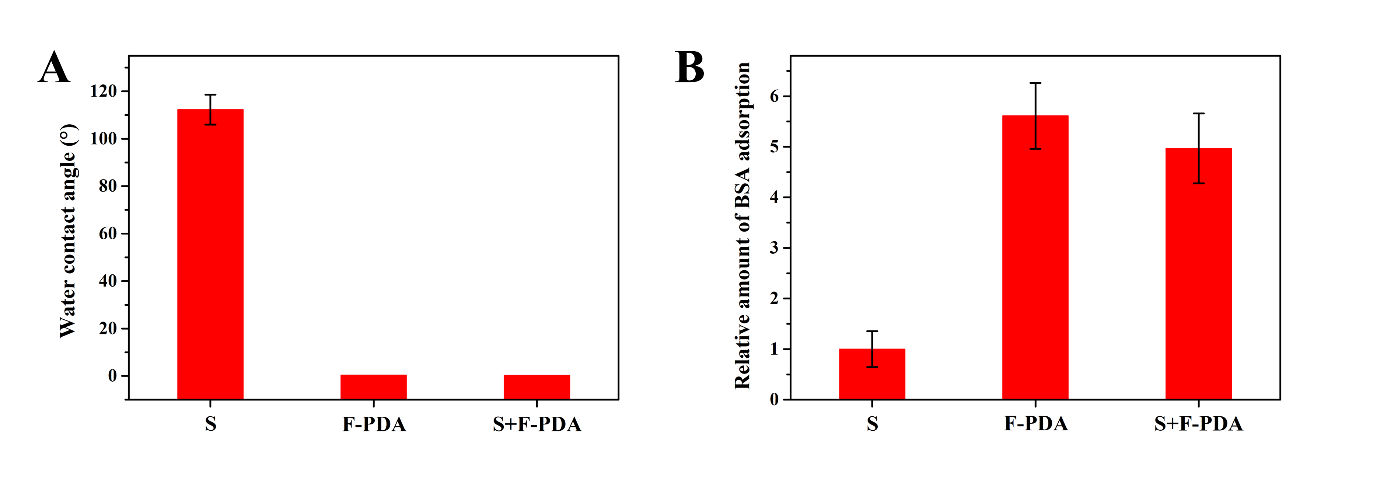


Figure S9 (A) Water contact angle and (B) relative amount of BSA adsorption by S, F-PDA, and bilayer S+F-PDA scaffolds.


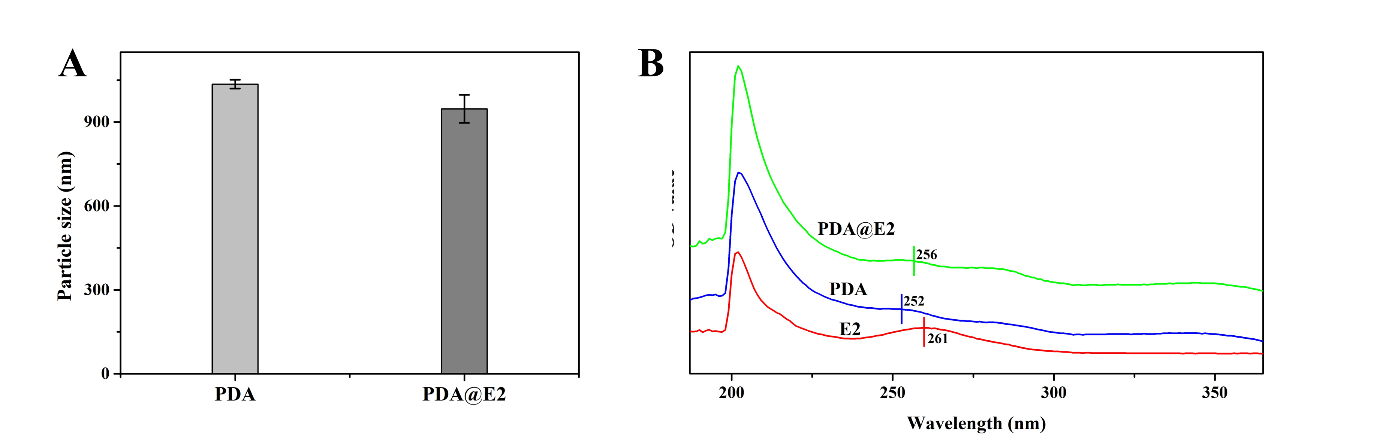


Figure S10 (A) Average particle size and (B) UV-vis spectra of PDA and PDA@E2 particles.


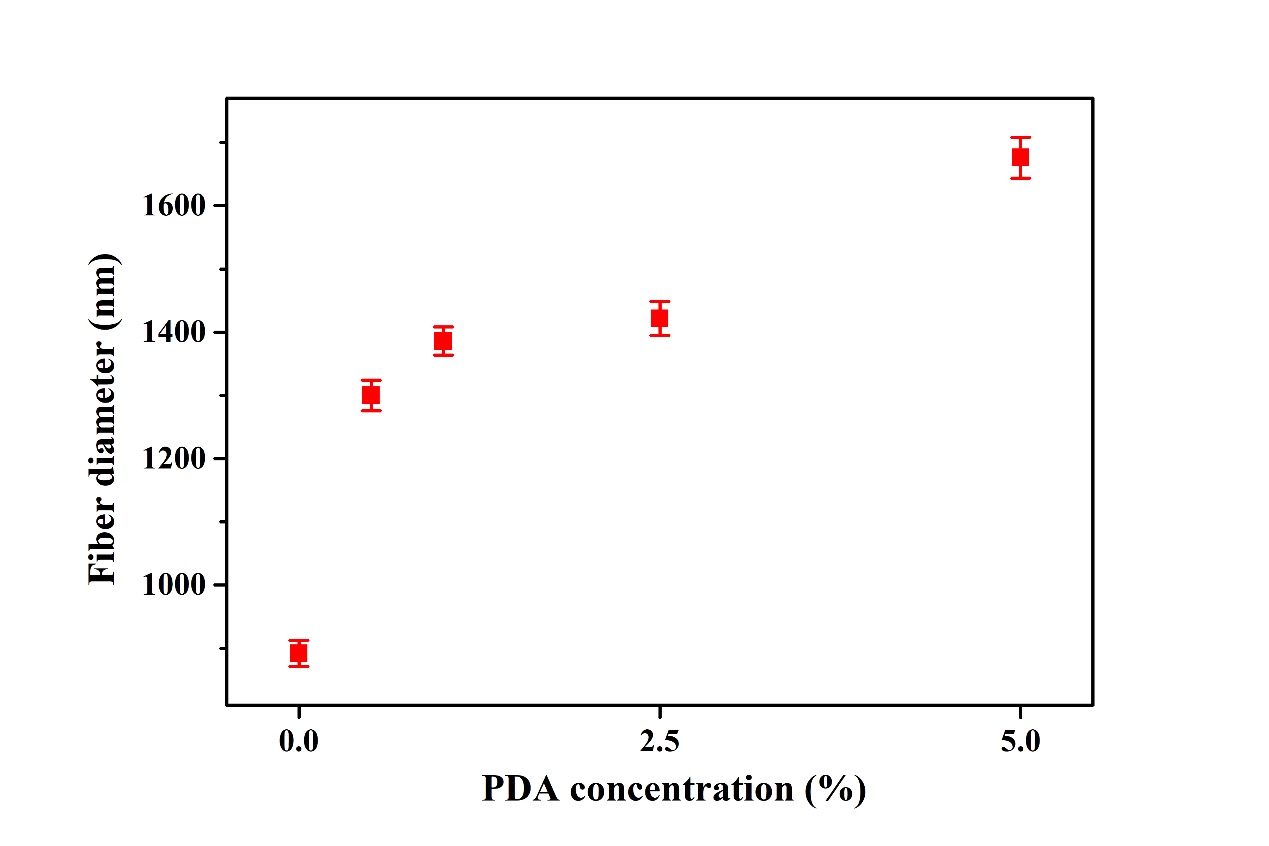


Figure S11 Average fiber diameter of electrospun PLGA/GelMA fibers having different concentrations of PDA particles.


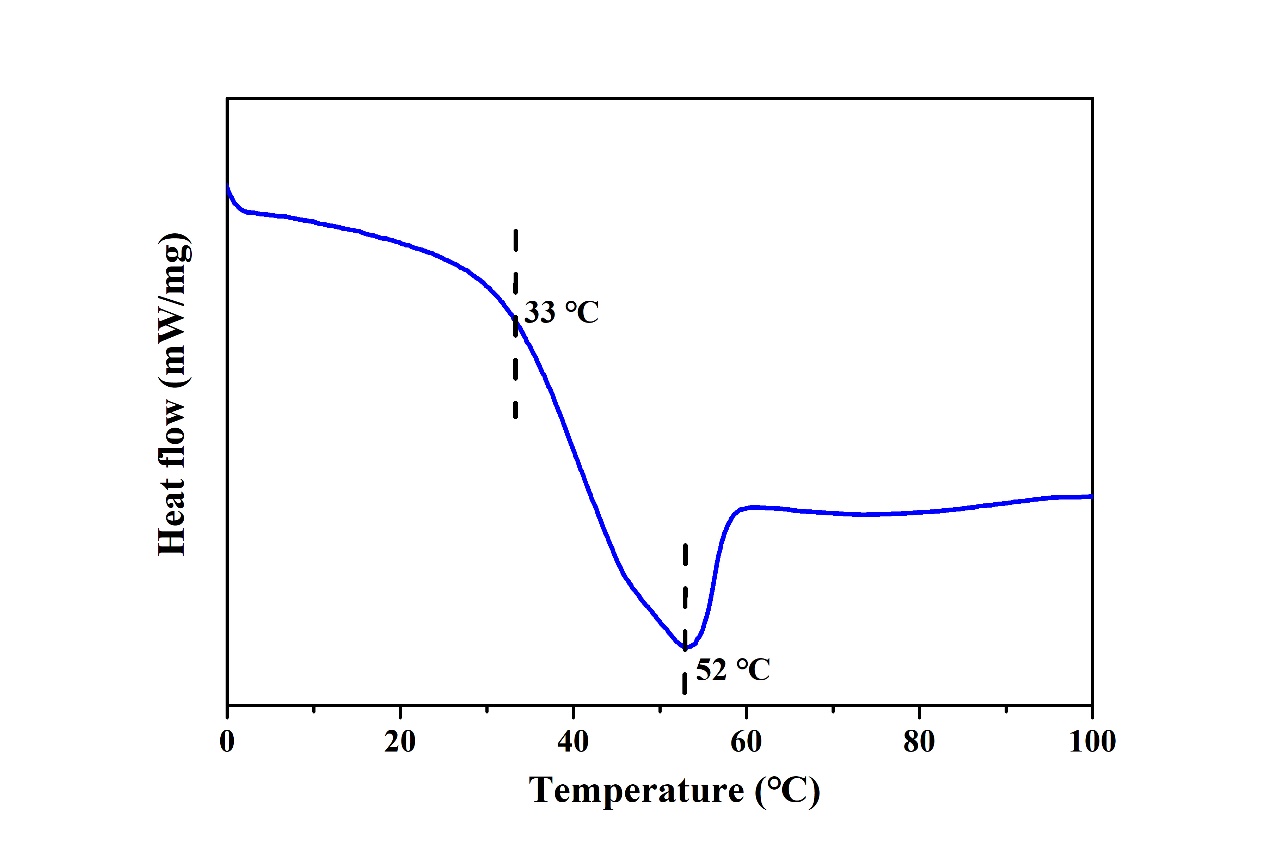


Figure S12 DSC curve of PLGA.


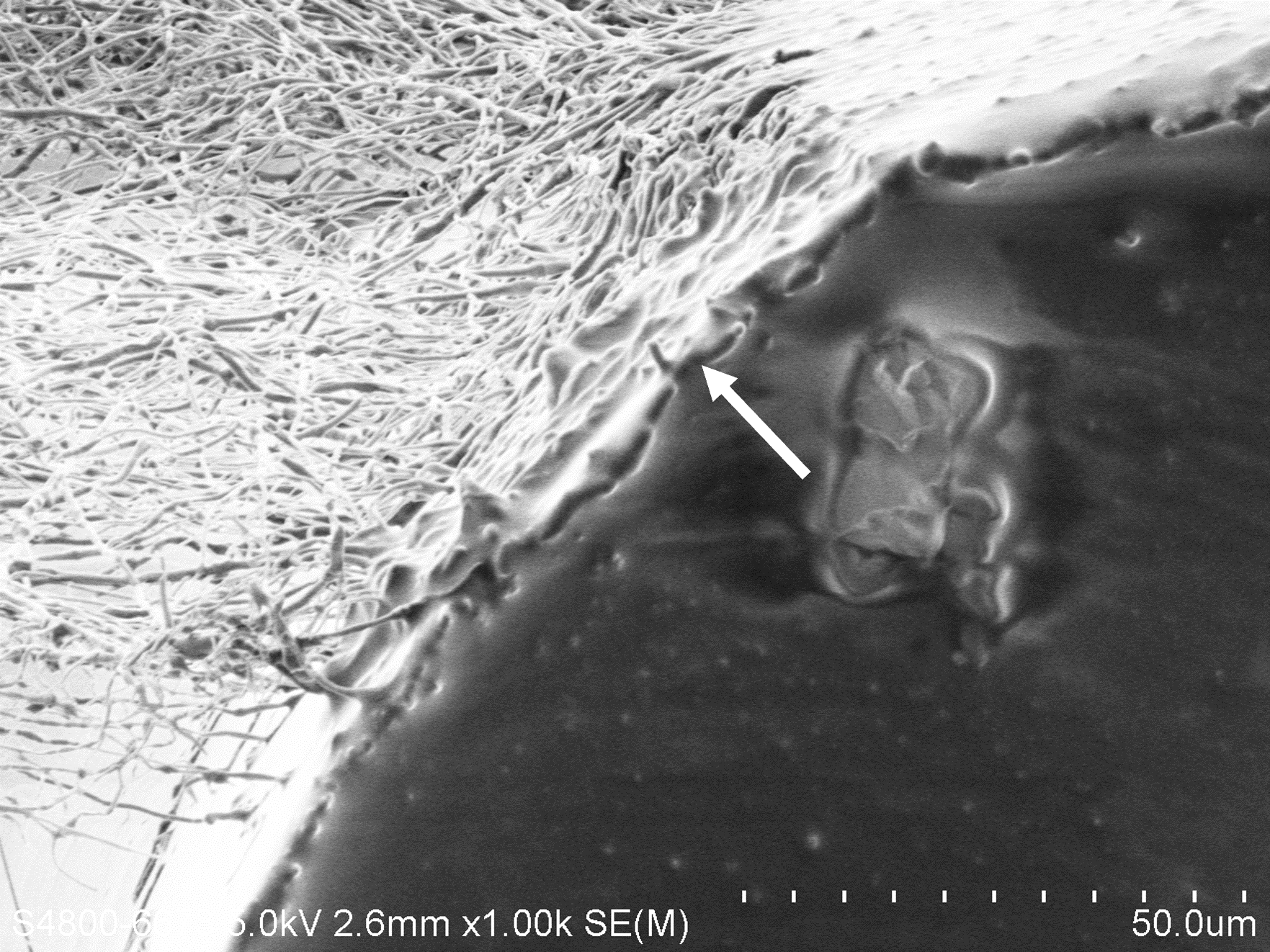


Figure S13 An SEM image providing the cross-sectional view of a bilayer scaffold at the initial electrospinning stage.


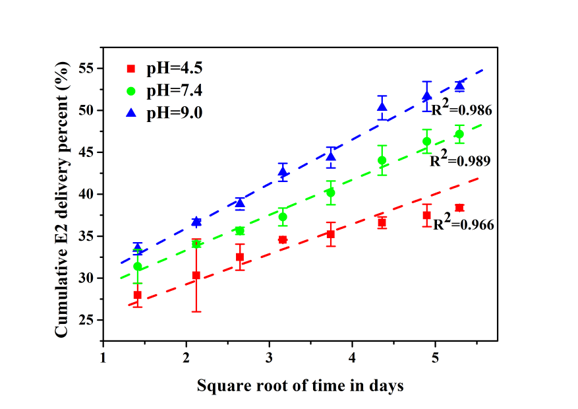


Figure S14 Analysis of E2 release using the Higuchi model for S+F-PDA@E2 scaffolds in pH 4.5, 7.4, and 9.0 environments.


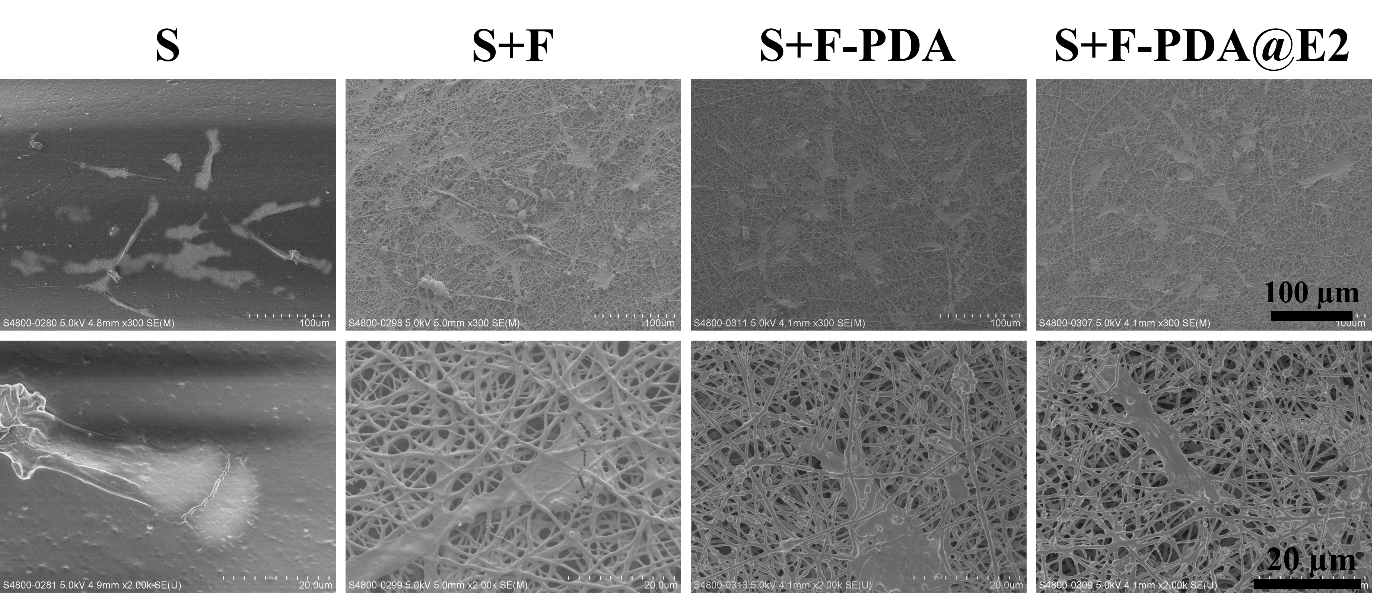


Figure S15 SEM images showing BMSC morphology on S, S+F, S+F-PDA and S+F-PDA@E2 scaffolds after 1-day culture.


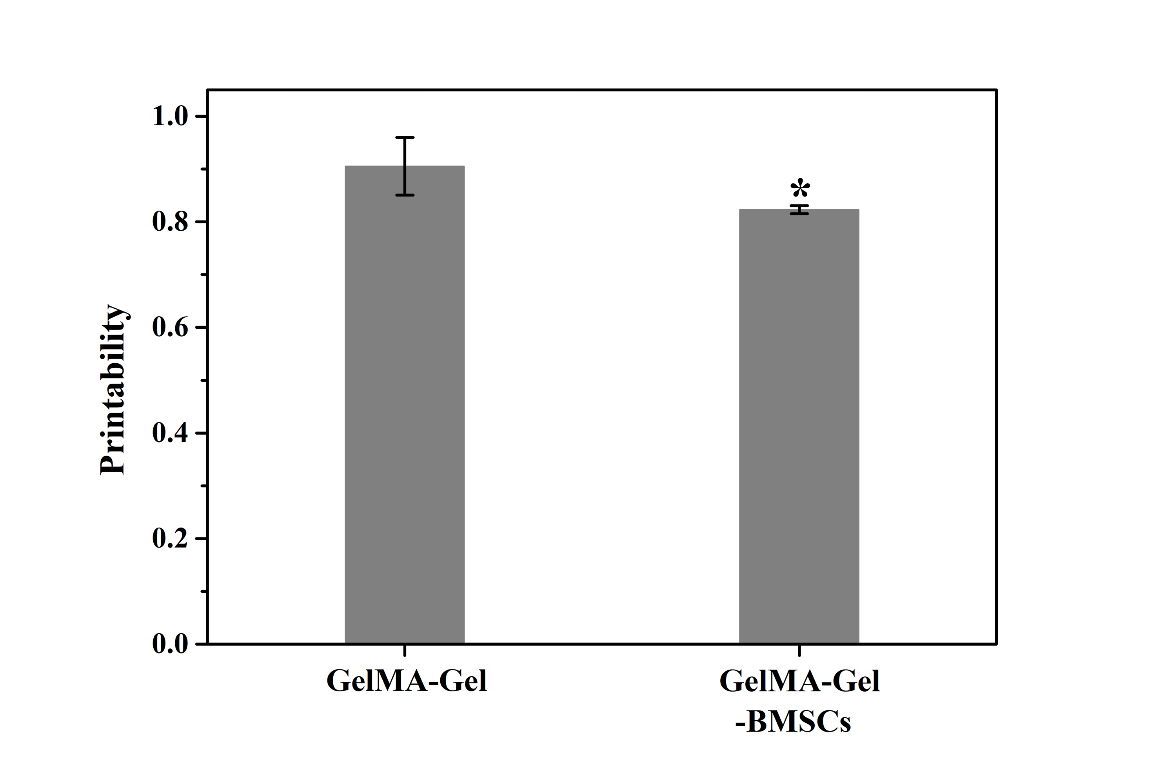


Figure S16 Printability of GelMA-Gel inks and GelMA-Gel-BMSC bioinks.

Table S1 Nominal and real percentages of PTMC in PTMC/TPU scaffolds.

| Scaffold | PTMC nominal percentage (%) | PTMC real percentage (%) |
| --- | --- | --- |
| PTMC:TPU=0.25:1 | 20.0 | 22.8 |
| PTMC:TPU=0.5:1 | 33.3 | 38.9 |
| PTMC:TPU=1:1 | 50.0 | 57.0 |
| PTMC:TPU=2:1 | 66.7 | 67.6 |

Table S2 Tensile properties of S+F-PDA and S+F-PDA@E2 bilayer scaffolds.

|  | Elastic strain (%) | Elastic strength (MPa) | Elastic modulus (MPa) | Ultimate strength (MPa) | Plastic strain (%) | Failure strength (MPa) |
| --- | --- | --- | --- | --- | --- | --- |
| S+F-PDA | 7.91 ± 1.50 | 0.25 ± 0.04 | 4.86 ± 0.40 | 0.60 ± 0.06 | 315.54 ± 57.47 | 0.44 ± 0.02 |
| S+F-PDA@E2 | 7.35 ± 1.13 | 0.23 ± 0.06 | 4.66 ± 0.38 | 0.61 ± 0.10 | 337.44 ± 38.96 | 0.42 ± 0.08 |
